# Silent yet impaired: Hidden memory processing deficits in asymptomatic individuals with moderate‐to‐severe white matter hyperintensities

**DOI:** 10.1002/alz.71084

**Published:** 2026-01-07

**Authors:** Aonan Li, Lei Liu, Yueyan Bian, Yuanyuan Lu, Xiuqin Jia, Yuanyuan Chen, Yi Tang, Yi Xing

**Affiliations:** ^1^ Department of Neurology & Innovation Center for Neurological Disorders Xuanwu Hospital, Capital Medical University, National Center for Neurological Disorders Beijing China; ^2^ Department of Radiology, Beijing Chaoyang Hospital Capital Medical University Beijing China; ^3^ National Center for Neurological Disorders Neurodegenerative Laboratory of Ministry of Education of the People's Republic of China Beijing China

**Keywords:** fractional anisotropy, neural oscillations, phase–amplitude coupling, visual short‐term memory, white matter hyperintensities

## Abstract

**INTRODUCTION:**

White matter hyperintensities (WMHs) are strongly associated with cognitive decline and dementia, but whether asymptomatic individuals with moderate‐to‐severe WMHs (msWMHs) are truly cognitively intact, particularly in visual short‐term memory (VSTM), remains unclear.

**METHODS:**

Electroencephalography and diffusion tensor imaging (DTI) was obtained from 45 msWMH patients and 38 normal controls during an occluded‐face delay‐matching task.

**RESULTS:**

Individuals with msWMH showed systematic deficits in face‐memory processing across encoding, maintenance, and retrieval, accompanied by reduced theta power and synchronization, altered alpha power, and disrupted theta–gamma coupling. DTI further revealed microstructural damage, particularly in the inferior fronto‐occipital fasciculus (IFOF), superior longitudinal fasciculus (SLF), and inferior longitudinal fasciculus (ILF), which mediated the effects on global cognition via encoding‐related theta oscillations.

**DISCUSSION:**

These findings indicate that individuals with msWMH already show hidden VSTM deficits. Theta oscillations mediate the link between tract integrity and global cognition, highlighting a potential preclinical intervention target.

**Highlights:**

- Asymptomatic individuals with moderate‐to‐severe white matter hyperintensities (msWMHs) already show visual short‐term memory (VSTM) deficits.
- Electroencephalography revealed abnormal neural oscillations during encoding, maintenance, and retrieval stages.
- Damage to the inferior fronto‐occipital fasciculus (IFOF), superior longitudinal fasciculus (SLF), and inferior longitudinal fasciculus (ILF) affects cognition via theta oscillations.
- Theta activity mediates structure ‐ function coupling and may serve as a preclinical intervention target.

## 1 BACKGROUND

Cerebral small vessel disease (CSVD) is a leading cause of vascular cognitive impairment and the second most common contributor to dementia.^1^, ^2^ Despite its clinical significance, CSVD typically progresses silently and lacks reliable and diverse early biomarkers. White matter hyperintensities (WMHs), the core imaging feature of CSVD, are widely recognized as an independent risk factor for cognitive impairment.^3^, ^4^, ^5^ Studies have shown a dose‐dependent association between WMH burden and cognitive decline, with greater WMH burden conferring increased dementia risk.^1^, ^4^ Yet, substantial heterogeneity exists between WMH burden and clinical phenotype: some individuals preserve cognition despite extensive WMHs.^6^, ^7^ Such heterogeneity suggests that conventional neuropsychological tests may lack sensitivity to subtle WMH‐related deficits and raises the critical question of whether asymptomatic individuals with moderate‐to‐severe WMHs (msWMHs) are truly free of cognitive impairment. Given the critical role of white matter pathways in rapid information transfer and network coordination, their disruption may preferentially compromise short‐term memory processes that depend on precise temporal integration. Visual short‐term memory (VSTM), a core component of cognitive processing,^8^ is critical for higher‐order functions and may serve as an early marker of CSVD‐related cognitive decline. Studying VSTM in individuals with msWMHs could uncover early neurophysiological alterations, identify potential intervention targets, and inform strategies to prevent and reduce dementia risk.

VSTM involves three stages: encoding, retention, and retrieval.^9^, ^10^ To investigate its dynamic processes and underlying mechanisms, we employed an occluded‐face delayed‐match‐to‐sample (OFDM) task combined with electroencephalography (EEG) recording.^10^ Face encoding and retrieval rely on neural processes supporting early detection (P1), structural representation (N170), and higher‐order configural analysis (P2),^11^, ^12^ whereas memory maintenance is reflected in posterior negative slow wave (NSW) that scales with the amount of retained visual information.^13^, ^14^ These event‐related potentials (ERPs) are typically accompanied by changes in neural oscillations. Electrophysiological evidence indicates that short‐term memory relies on specific oscillatory dynamics: theta rhythms structure the encoding and sequential organization of information, whereas alpha rhythms support maintenance by regulating attention and inhibiting irrelevant activity.^15^, ^16^, ^17^ Moreover, successful memory representation relies on precise theta–gamma coupling.^18^ However, no studies to date have systematically examined short‐term memory processing and its oscillatory alterations in individuals with msWMHs.

Neural oscillation and phase synchronization underlying VSTM depend on the integrity of white matter tracts, which enable inter‐regional information transfer and play a critical role in maintaining communication across distributed brain regions.^9^ Previous studies have shown that VSTM processing involves long‐range white matter tracts, such as the inferior fronto‐occipital fasciculus (IFOF), the superior longitudinal fasciculus (SLF), and the inferior longitudinal fasciculus (ILF).^9^ Whether memory deficits in asymptomatic individuals with msWMHs are accompanied by microstructural damage in these memory‐related tracts has not yet been investigated. Moreover, the potential link between the microstructure of these tracts and neural oscillations during memory encoding remains largely unexplored.

In summary, we aimed to investigate the temporal dynamics and oscillatory mechanisms of short‐term memory deficits in patients with msWMHs using an OFDM task combined with EEG. Diffusion tensor imaging (DTI) was used to extract fractional anisotropy (FA) as a measure of white matter tract integrity, enabling examination of how these tracts modulate memory processing and global cognition via theta rhythms. We hypothesized that: (1) VSTM deficits in msWMHs would reflect diminished neural dynamics during encoding, maintenance, and retrieval—indexed by P1, N170, P2, and NSW—primarily due to reduced theta and alpha oscillations during encoding/maintenance, as well as impaired cross‐frequency coupling throughout all stages; (2) these deficits would be accompanied by microstructural alterations in memory‐related tracts such as the IFOF, SLF, and ILF; and (3) impaired tract integrity may compromise transmission of occipital information to medial temporal and prefrontal networks, thereby disrupting local and inter‐regional oscillations (e.g., theta and alpha) and leading to memory and cognitive deficits. By integrating structural and functional measures, this study enhances understanding of VSTM impairment in asymptomatic msWMHs, providing a theoretical basis for early diagnosis and intervention in CSVD.

## 2 METHODS

### 2.1 Participants

This study enrolled a total of 83 participants, comprising 45 individuals with clinically silent msWMHs and 38 normal controls (NCs) who had no or mild WMHs (Fazekas Grade 0–1) and no cognitive impairment. All participants were recruited between March 2024 and April 2025, from the White Matter Degeneration Clinic at Xuanwu Hospital, Capital Medical University. All NCs were recruited from the Baizhifang community in Beijing and were matched with the WMH group in terms of age, sex, and education. They self‐reported no cognitive decline, and their neuropsychological test scores fell within the normal range. All participants underwent comprehensive clinical and cognitive assessments, including medical history review, neurological examination, and neuropsychological testing. The inclusion and exclusion criteria for patients with msWMHs are detailed in the Supplementary Material. Written informed consent was obtained from all participants or their legal guardians, and the study protocol was approved by the Ethics Committee of Xuanwu Hospital, Capital Medical University (No. 2024092).

### 2.2 Neuropsychological assessment

Cognitive function was assessed across multiple domains using neuropsychological tests. Global cognition was evaluated using the Montreal Cognitive Assessment (MoCA). Memory performance was measured using the World Health Organization–University of California Los Angeles (WHO‐UCLA) Auditory Verbal Learning Test (AVLT) and the Digit Span Test, forward and backward (DST‐F/B). Attention and executive function were evaluated with the Trail Making Test (TMT), whereas visuospatial ability was examined using the Clock Drawing Test (CDT). Language function was assessed with the Boston Naming Test (BNT). In addition, anxiety and depressive symptoms were evaluated using the Hamilton Anxiety Scale (HAMA) and the Hamilton Depression Scale (HAMD).

**RESEARCH IN CONTEXT**

1. **Systematic review**: A PubMed search revealed that white matter hyperintensities (WMHs), the core imaging feature of cerebral small vessel disease, are common in aging and predict dementia risk. Prior studies link WMHs to processing speed and executive dysfunction, but few have examined visual short‐term memory (VSTM) and its neural oscillatory basis. Evidence for structure–function coupling between white matter microstructure and oscillatory activity in asymptomatic WMH individuals is lacking.
2. **Interpretation**: This study demonstrates that asymptomatic individuals with moderate‐to‐severe WMHs already exhibit VSTM impairment, characterized by abnormal electroencephalography oscillations and microstructural damage to key white matter tracts. Theta oscillations mediate the association between tract integrity and global cognition, providing a mechanistic explanation for hidden memory deficits.
3. **Future directions**: Future longitudinal studies should investigate whether oscillatory biomarkers predict cognitive decline in WMH populations and assess their potential as targets for noninvasive neuromodulation or preventive interventions.

### 2.3 Occluded‐face delayed‐match ‐ to ‐ sample (OFDM) paradigm

**FIGURE 1:**
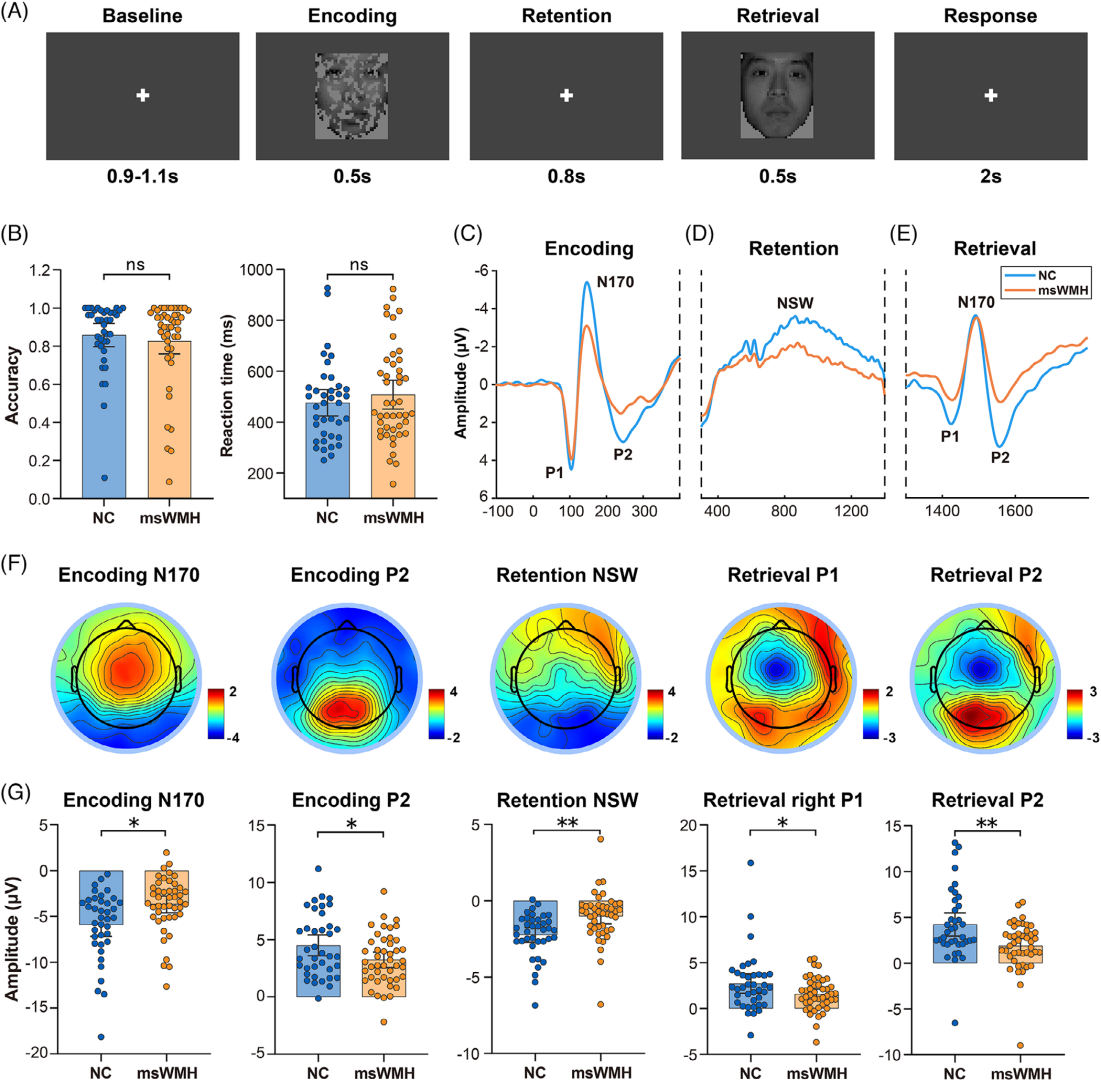
Behavioral and ERP results during the occluded‐face delayed‐match ‐ to ‐ sample (OFDM) task in NCs and msWMH individuals. (A) OFDM: Two face images are presented sequentially—an occluded face followed by an intact one. Each image is presented for 500 ms, followed by an 800 ms blank screen with a central fixation cross as the inter‐stimulus interval (ISI). Participants indicate whether the two images depict the same face by pressing the “left” key (match) or the “right” key (mismatch) within 2000 ms after the second image. An inter‐trial interval (ITI) of 1000 ms was applied between trials. (B) Behavioral performance (accuracy and reaction time) of the OFDM task in both groups. (C) The posterior P1, N170, and P2 components were evoked by occluded faces during the encoding stage in both groups. (D) The posterior NSW component was evoked during the retention stage in both groups. (E) The posterior P1, N170, and P2 components were evoked by the intact faces during the retrieval stage in both groups. (F) Topography of the five ERP components corresponding to encoding‐related N170, P2, maintenance‐related NSW, and retrieval‐related P1, P2 components. G) Bar charts of the five ERP components across the two groups. msWMH, moderate‐to‐severe white matter hyperintensity; NC, normal control; NSW, negative slow wave. ** for *p* < 0.01, * for *p* < 0.05, and ns for not significant.

The OFDM task was employed to examine visual short‐term memory (Figure 1A). Ninety neutral facial images (45 male and 45 female) were randomly selected from the Chinese Standard Emotional Facial Expression Image Database. All images were partially occluded using Adobe Photoshop to generate occluded faces, with an average occlusion rate of 41.5%.

As illustrated in Figure 1A, the experimental procedure was as follows: each trial consisted of two facial images presented sequentially on an LCD monitor using E‐Prime 2.0 software. The first stimulus was an occluded face, followed by its corresponding complete face. Each image was presented for 500 ms, separated by an interstimulus interval (ISI) of 800 ms consisting of a blank screen with a central fixation cross. Participants were instructed to judge whether the two images showed the same face and to respond within 2000 ms after the onset of the second image by pressing the left key (“match”) or the right key (“non‐match”). The intertrial interval (ITI) was set at 1000 ms. The formal experiment began with 10 practice trials, followed by 80 formal trials, to ensure that participants fully understood the task requirements and were familiar with the procedure.

### 2.4 EEG recording and preprocessing

EEG data were recorded during the OFDM task in an acoustically shielded room. Continuous data were collected using a 128‐channel EGI system (Electrical Geodesics Inc., USA) at a sampling rate of 1000 Hz, with Cz as the online reference. Electrode impedance was maintained below 50 kΩ throughout the recording.

The data were preprocessed using EEGLAB v2023.1 in the MATLAB environment (Mathworks, Natick, MA, USA).^19^ First, EEG data were band‐pass filtered between 0.1 and 75 Hz and notch filtered at 50 Hz using an finite impulse response (FIR) filter to reduce line noise. Then, the signals were downsampled to 500 Hz and visually inspected. Trials contaminated by large movements, muscle activity, drifts, or abrupt jumps were removed. Ocular and other periodic artifacts were corrected using independent component analysis (ICA).^19^ After artifact correction, the data were re‐referenced to the common average. Preprocessed continuous data were segmented into epochs from −200 ms to 1800 ms relative to stimulus onset, with baseline correction applied using the −200 to 0 ms interval. Epochs were further screened using a sliding‐window peak‐to‐peak method (window length: 200 ms; step size: 100 ms; threshold: ± 100 µV),^20^ and trials exceeding this threshold were discarded. Finally, epochs were averaged according to experimental conditions.

### 2.5 Event‐related potential (ERP) analysis

ERP waveforms were obtained by averaging the epochs corresponding to the occluded and intact face stimuli. During the encoding and retrieval stages, P1, N170, and P2 components were elicited, whereas a posterior negative slow wave (or NSW) was observed during the memory retention stage. Based on the grand‐averaged waveforms, scalp topographies, and previous studies,^12^, ^14^, ^21^ we selected encoding‐related components elicited by occluded faces for comparison: the peak amplitudes of the P1 component (90–120 ms) in the occipital region (averaged across E71, E72, E75, E76, E77, E83, E84, E85, E90, E91, and E96), the N170 component (140–190 ms) in the temporo‐occipital region (averaged across E69, E70, E71, E76, E83, E89, E74, E75, and E82), and the P2 component (220–270 ms) in the occipital region (averaged across E66, E67, E71, E72, E76, E77, and E84). For the retention stage, the mean amplitude of the posterior (averaged across E71, E76, and E77) NSW (950–1200 ms) was used as the index of face‐memory maintenance. For the retrieval stage, we selected components elicited by intact faces: the peak amplitude of P1 (100–140 ms) at bilateral occipital sites (averaged across E58, E59, E65, E66, and E70 for left P1, averaged across E84, E91, E96, E97, E90, and E101 for right P1), and the peak amplitudes of N170 (190–230 ms) at the right temporal‐occipital region (averaged across E89, E95, E96, and E100) and P2 (230–280 ms) at occipital region (averaged across E65, E69, E70, E74, and E76).

### 2.6 Time–frequency analysis (TFA)

Preprocessed EEG data were further downsampled to 250 Hz and segmented into epochs from −1000 ms to 3000 ms relative to stimulus onset. Trials with amplitude fluctuations exceeding ± 100 µV were excluded using the sliding‐window peak‐to‐peak method. Time–frequency analysis (TFA) was then applied to compute event‐related spectral perturbation (ERSP) and inter‐trial phase coherence (ITPC, i.e., phase synchronization). The time–frequency decomposition was calculated on the single trial from 3 to 45 Hz by the newtimef function of EEGLAB v2023.1 under Matlab 2023b. The number of cycles for Morlet wavelets varied with the frequency (3 cycles at 3 Hz to 22.5 cycles at 45 Hz, in a step of 0.5 Hz). The resulting time–frequency data were averaged across trials and baseline‐corrected using the −400 to −200 ms interval.

Based on the grand‐averaged time–frequency and topographical distribution, as well as previous studies,^22^, ^23^, ^24^ theta (4–7 Hz, 0–400 ms) power in the frontal‐central (averaged across E5, E6, E7, E12, E13, E106, and E112) and occipital (averaged across E70, E71, E75, E76, E83, E84, E90, E91, and E96) areas were selected as the regions of interest (ROIs) during the encoding stage; alpha (9–14 Hz, 1000–1200 ms) power in the right occipital (averaged across E90, E91, E96, E97, and E101) region was selected as the ROI during the retention stage; theta (4‐7 Hz, 1500–1700 ms) power in the frontal‐central (averaged across E11, E12, E18, E19, and E20) area, and theta (4‐7 Hz, 1350–1650 ms) power in the left occipital (averaged across E66, E67, E70, E71, and E72) and right occipital (averaged across E83, E84, E90, E91, and E96) areas were selected as ROIs during the retrieval stage. We also extracted theta‐band (4–7 Hz) ITPC during the encoding stage (50–400 ms) from occipital electrodes (averaged across E66, E67, E70, E71, E72, E75, and E76), and during the retrieval stage (1350–1600 ms) from right occipital (averaged across E76, E83, E84, E90, E91, and E96) regions for statistical comparisons.

### 2.7 Phase–amplitude coupling (PAC)

Memory encoding and retrieval rely on the modulation of gamma power by the phase of ongoing theta oscillations.^18^ Disruptions in theta activity are paralleled by abnormalities in theta–gamma phase–amplitude coupling (PAC). To quantify PAC between theta and gamma oscillations, we employed the Kullback–Leibler Modulation Index (KLMI) for each memory stage (i.e., encoding, maintenance, and retrieval).^25^ EEG signals were band‐pass filtered into theta (4–7 Hz), low gamma (31–45 Hz), and high gamma (55–70 Hz) frequency ranges using zero‐phase FIR filters. The analytic theta phase ϕ(t) and gamma amplitude envelope A(t) were extracted using the Hilbert transform.

For each trial, channel, and task stage (encoding: 0–0.5 s, retention: 0.5–1.3 s, and retrieval: 1.3–1.8 s), the gamma sub‐band amplitude values were binned according to the theta phase (divided into *N = 18* equal phase bins of 20^°^). The normalized amplitude distribution was then defined as:

$$P\;\left(j\right)=\frac{A{\left(t\right)}_{\phi \left(t\right)\in \mathrm{bin}\;j}}{\sum_{k=1}^{N}A{\left(t\right)}_{\phi \left(t\right)\in \mathrm{bin}\;j}}\;,\;j\;=\;1,\cdots ,\;N$$

where ${<A(t)>}_{\phi (t)\in \mathrm{binj}}$ denotes the mean gamma amplitude within the j‐th phase bin. *p* (j) represents the normalized probability of observing gamma amplitude in the j‐th theta phase bin.

The strength of PAC was quantified using the KLMI, defined as:

$$KLMI=\frac{D_{\mathrm{KL}}\left(P||U\right)}{\log\left(N\right)}\;,D_{\mathrm{KL}}\left(P|\;|U\right)=\sum_{j=1}^{N}P\left(j\right)\log\frac{P\left(j\right)}{U\left(j\right)}$$

where $D_{\mathrm{KL}}\left(P||U\right)$ is the Kullback–Leibler divergence between the observed distribution *p* (j) and the uniform distribution *U* (j). The normalization by *log* (N) bounds KLMI between 0 and 1, with higher values indicating stronger PAC.

To control for spurious coupling, we applied a surrogate data procedure in which 200 surrogate datasets were generated by randomly shifting the theta phase time series relative to the gamma amplitude.^25^, ^26^ The distribution of surrogate KLMI values served as the null model, against which observed KLMI values were standardized to yield z‐scores. These KLMI z‐scores were calculated at each channel and averaged across trials within each memory stage. Based on previous findings, the KLMI z‐scores in frontal and left parietal regions during encoding,^27^, ^28^ left temporo‐parietal region during retention,^22^, ^29^ and left frontal region during retrieval were selected for subsequent group‐level statistical analyses.^30^, ^31^

### 2.8 MRI data acquisition

MRI scans were conducted on a 3.0 T Siemens scanner (Magnetom Spectra, Siemens Healthineers, Erlangen, Germany). Structural T1‐weighted images were acquired using a three‐dimensional (3D) magnetization‐prepared rapid gradient echo (MPRAGE) protocol: repetition time (TR) = 2300 ms, echo time (TE) = 2.19 ms, inversion time (TI) = 900 ms, flip angle = 8^°^, field of view (FOV) = 256 × 256 mm^2^, voxel size = 1 × 1 × 1 mm^3^, and 176 slices. Fluid‐attenuated inversion recovery (FLAIR) imaging was performed with distinct parameters (TR = 5000 ms, TE = 390 ms, TI = 1800 ms, FOV = 240 × 240 mm^2^, voxel size = 0.9 × 0.9 × 1 mm^3^). DTI was performed using a single‐shot spin ‐ echo echo ‐ planar imaging (SE‐EPI) sequence (TR = 4300 ms, TE = 99 ms, FOV = 256 × 256 mm^2^, voxel size = 2 mm^3^, 60 slices, 64 directions, *b* = 0 and 1000 s/mm^2^, bandwidth = 1396 Hz/Px). Quantitative susceptibility mapping (QSM) was acquired with a 3D multi‐echo gradient‐echo sequence (FOV = 220 × 220 mm^2^, voxel size = 0.9 × 0.9 × 2 mm^3^, 64 slices, TR = 50 ms, eight echo times: 6.15‐45 ms, flip angle = 15^°^, bandwidth = 260 Hz/Px).

### 2.9 MRI data processing

WMHs were segmented using the Lesion Prediction Algorithm (LPA) implemented in the LST toolbox (https://www.statistical‐modeling.de/lst.html), based on FLAIR images with T1‐weighted images as anatomic references. LPA is based on a logistic regression model trained on data from 53 patients with multiple sclerosis (MS), where binary lesion maps served as response variables. As covariates, the model incorporates a lesion belief map and a spatial covariate accounting for voxel‐specific lesion probability. Large‐scale regression fitting was performed using Regression HD (http://www.applied‐statistics.de/RegressionHD_en.html). The fitted model parameters were then applied to new images to estimate lesion probability at each voxel. To evaluate performance, LPA‐derived probability maps (thresholded at 0.5) were compared against manual lesion masks in FLAIR space. Finally, WMH volumes were normalized to total intracranial volume (TIV) to control for interindividual differences in brain size. Lacunes were defined as round or ovoid cavities with cerebrospinal fluid (CSF)–like signal measuring 3–20 mm in diameter. Cerebral microbleeds (CMBs) were identified on QSM images as small (2–10 mm), well‐demarcated hypointense lesions. Enlarged perivascular space (EPVS) burden was quantified using a validated deep learning–based multimodal segmentation approach integrating both T1‐ and T2‐weighted MRI; full methodological details are provided in the Supplementary Materials. All CSVD markers were evaluated by two trained, blinded radiologists in accordance with standards for reporting vascular changes on neuroimaging (STRIVE),^32^ and discrepancies were resolved by a senior neurologist.

DTI underwent preprocessing using the FMRIB Software Library (FSL; http://www.fmrib.ox.ac.uk/fsl/), including denoising, correction for susceptibility‐induced distortion, eddy currents, and head motion. Diffusion tensors and scalar diffusion metrics, including FA, were then calculated using the DTIFIT module in FSL. FA maps were spatially normalized to the FMRIB‐1 mm‐FA standard template via nonlinear registration using FNIRT. Regional diffusion metrics were extracted by mapping the HCP‐842 tractography atlas (http://dsi‐studio.labsolver.org/download‐images/hcp‐842‐template) to obtain ROI‐based FA values. Based on previous studies,^33^, ^34^, ^35^, ^36^ we extracted FA values from memory‐related white matter tracts, including the fornix (FX), corpus callosum (CC), arcuate fasciculus (AF), cingulum (CG), IFOF, SLF, ILF, and uncinate fasciculus (UF) for further analysis.

### 2.10 Statistical analysis

Data analysis was performed with SPSS 26.0 (IBM Corp., Armonk, NY, USA) and R (version 4.3.3). Normality was assessed using the Shapiro–Wilk test, and homogeneity of variance was assessed using Levene's test. Independent sample *t*‐tests were used to compare continuous variables, including demographics, neuropsychological assessments, behavioral performance (accuracy and reaction time), EEG metrics, and MRI measures, between the NC and msWMH groups. Chi‐square tests were used to compare categorical variables. Categorical variables are presented as percentages, and continuous variables are presented as means ± SDs.

A linear regression model was established to explore the relationships between EEG measures, imaging measures, and clinical indicators. Age, sex, and education were included as confounding variables for adjustment. Furthermore, mediation regression models were used to investigate the mediating role of neural oscillations between FA and cognitive performance, while controlling for the above‐mentioned covariates. To ensure the robustness of the findings, bootstrap techniques were applied in both the linear regression and mediation models, generating 2000 bootstrap samples to derive 95% confidence intervals (CIs). After identifying EEG metrics associated with memory processing deficits in patients with msWMHs, logistic regression and receiver‐operating characteristic (ROC) analyses were conducted to evaluate the diagnostic accuracy of EEG metrics for varying degrees of white matter damage, with odds ratios (ORs), 95% CIs, and areas under the curve (AUCs) calculated. A significance level of *p*< 0.05 (two‐tailed) was applied for all statistical tests. Group‐wise comparisons of white matter tract metrics between NC and msWMH groups were false discovery rate (FDR) corrected to control for multiple comparisons.

## 3 RESULTS

### 3.1 Demographic information and behavioral performance

A total of 83 participants were included in the analysis, comprising 38 normal controls (NCs) and 45 patients with msWMHs. Detailed demographic, neuropsychological, and clinical data, including performance on the OFDM task, are presented in Table 1. There were no significant group differences in demographic information (i.e., age, sex, and education) and neuropsychological measures. There were also no significant group differences in accuracy and reaction time (RT) in the OFDM task (Accuracy: *t*_81_ = 0.651, *p* = 0.517; RT: *t*_81_ = –0.840, *p* = 0.403; Figure 1B and Table 1).

**TABLE 1:** Characteristics of participants.

	NC (*n* = 38)	msWMH (*n* = 45)	*p*
Demographics			
Age, years	65.4 (4.8)	66.1 (6.1)	0.573
Females, n (%)	28 (73.7)	32 (71.1)	0.261
Education, years	12.9 (2.3)	12.7 (2.5)	0.727
Neuropsychological test			
*OFDM task*			
Accuracy	0.858 (0.185)	0.828 (0.229)	0.517
Reaction time	475.737 (157.494)	508.114 (188.389)	0.403
*Global cognition*			
MMSE	28.8 (1.2)	28.6 (1.5)	0.509
MoCA	26.5 (1.8)	26.0 (1.7)	0.228
*Visual spatial ability*			
CDT	2.7 (0.5)	2.7 (0.5)	0.548
* Memory*			
AVLT‐Immediate recall	27.2 (4.6)	26.1 (4.6)	0.288
AVLT‐Delayed recall	10.6 (2.1)	9.9 (2.2)	0.165
AVLT‐Recognition recall	13.0 (1.6)	12.4 (1.5)	0.087
DST Forward	8.4 (0.7)	8.1 (0.9)	0.119
DST Backward	5.2 (1.2)	4.8 (1.2)	0.091
*Attention and executive function*			
TMT Parts B‐A	29.0 (25.0)	32.7 (24.4)	0.502
* Language proficiency*			
BNT	27.1 (1.7)	26.6 (1.5)	0.205
*Anxiety and depression*			
HAMA	3.9 (3.5)	3.6 (2.4)	0.601
HAMD	4.2 (4.4)	4.0 (3.0)	0.763
Brain MRI			
WMH	2.7 (1.3)	12.0 (10.5)	< 0.001

Continuous variables are presented as mean ± SD, and categorical variables as n (%). Group differences were compared using independent‐sample *t*‐tests for continuous variables and χ^2^ tests for categorical variables.

Abbreviations: AVLT, Auditory Verbal Learning Test; BNT, Boston Naming Test; CDT, Clock Drawing Test; DST, Digit Span Test; HAMA, Hamilton Anxiety Scale; HAMD, Hamilton Depression Scale; NC, normal control; MoCA, Montreal Cognitive Assessment; OFDM, occluded ‐face delayed ‐ match ‐ to ‐ sample; TMT, Trail Making Test; msWMH, moderate‐to‐severe white matter hyperintensity.

### 3.2 Short‐term memory deficits in patients with msWMHs

To evaluate the electrophysiological characteristics of VSTM in msWMHs, we analyzed ERP components during the encoding, retention, and retrieval stages. During the encoding stage, the msWMH group showed significantly reduced N170 and P2 peak amplitudes compared with NCs (N170: *t*_81_ = –2.968, *p* = 0.004; P2: *t*_81_ = 2.276, *p* = 0.026; Figure 1C,F,G). In the retention stage, msWMH individuals exhibited a lower posterior NSW amplitude (*t*_81_ = –2.781, *p* = 0.007; Figure 1D,F,G). During retrieval, they further showed reduced right occipital P1 (*t*_81_ = 2.031, *p* = 0.046; Figure 1E–G) and P2 (*t*_81_ = 3.275, *p *= 0.002; Figure 1E–G) peak amplitudes relative to NCs (see Table S1 for details). But there were no significant group differences in encoding‐related P1 and retrieval‐related left occipital P1 components (*t*_81_ ≤ 1.477, *p* ≥ 0.144; Figure S1 and Table S1). These results indicate that msWMH individuals exhibit abnormal neural dynamics during the encoding, retention, and retrieval stages of face memory.

### 3.3 Memory‐related neural oscillatory alterations in patients with msWMHs

**FIGURE 2:**
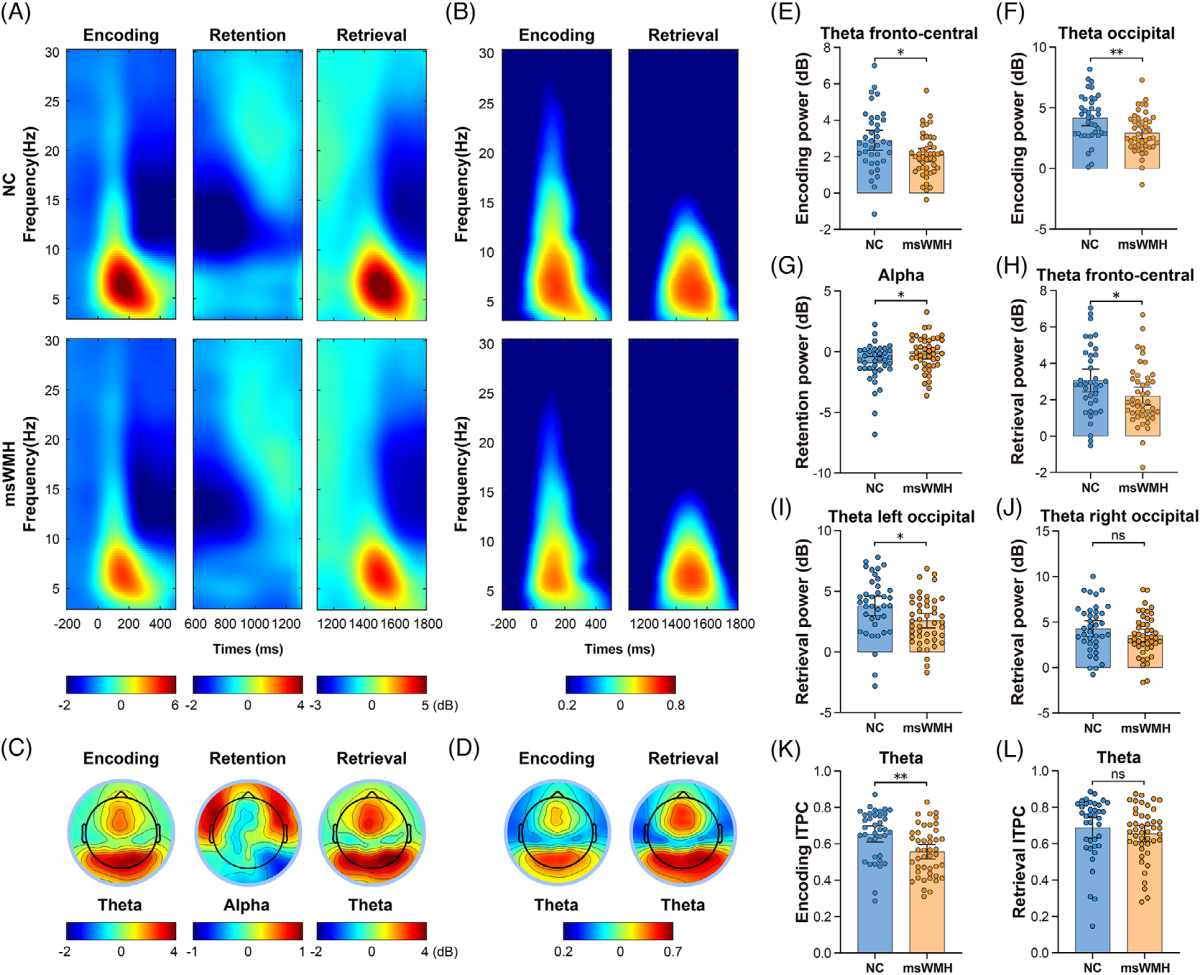
Comparisons of power and phase synchronization (i.e., ITPC) between the two groups during the encoding, retention, and retrieval stages. (A) Grand‐averaged event‐related spectral perturbations (ERSPs) in both groups during the encoding, retention, and retrieval stages. Theta power was clearly altered during the encoding and retrieval stages, whereas alpha power was altered during the retention stage. (B) Grand‐averaged inter‐trial phase coherence (ITPC) in both groups during the encoding and retrieval stages. Theta‐band ITPC was clearly altered during the encoding stage. (C) Grand‐averaged topographical distributions of the theta band power during the encoding and retrieval stages, and alpha power during the retention stage. (D) Grand‐averaged topographical distributions of theta band ITPC during the encoding and retrieval stages. (E–J) Bar charts showing group differences in fronto‐central and occipital theta band power across the encoding and retrieval stages, and alpha band power during retention. (K–L) Bar charts showing group differences in theta ITPC during encoding and retrieval stages. msWMH, moderate‐to‐severe white matter hyperintensity; NC, normal control. ** for *p* < 0.01, * for *p* < 0.05, and ns for not significant.

Neural dynamics in face memory are orchestrated by theta and alpha oscillations, which support encoding, retention, and retrieval.^37^, ^38^, ^39^, ^40^ We further examined the neural oscillations during face‐memory processing in patients with msWMHs. Patients with msWMHs exhibited reduced theta power in the fronto‐central (*t*_81_ = 2.623 *p* = 0.010; Figure 2E and Figure S2A, B) and occipital (*t*_81_ = 3.168, *p* = 0.002; Figure 2A,C,F) regions, and decreased theta phase synchronization in the occipital region (*t*_81_ = 3.297, *p* = 0.001; Figure 2B,D,K) during encoding; altered alpha power in the right occipital region during retention (*t*_81 _= –2.270, *p* = 0.026; Figure 2A,C,G); and reduced theta power in the fronto‐central (*t*_81_ = 2.198, *p* = 0.031; Figure 2H and Figure S2A, B) and left occipital (*t*_81_ = 2.516, *p* = 0.014; Figure 2A,C,I) but not right occipital regions (*t*_81_ = 1.408, *p* = 0.163; Figure 2A,C,J), and no change in theta phase synchronization (*t*_81_ = 0.937, *p* = 0.352; Figure 2B,D,L) during retrieval (see Table S2). These results suggest that impairment in face encoding, maintenance, and retrieval appears to be grounded in disrupted neural oscillatory dynamics, specifically reduced theta oscillation during encoding and retrieval, and altered alpha activity during retention.

### 3.4 Altered theta–gamma PAC in patients with msWMHs

**FIGURE 3:**
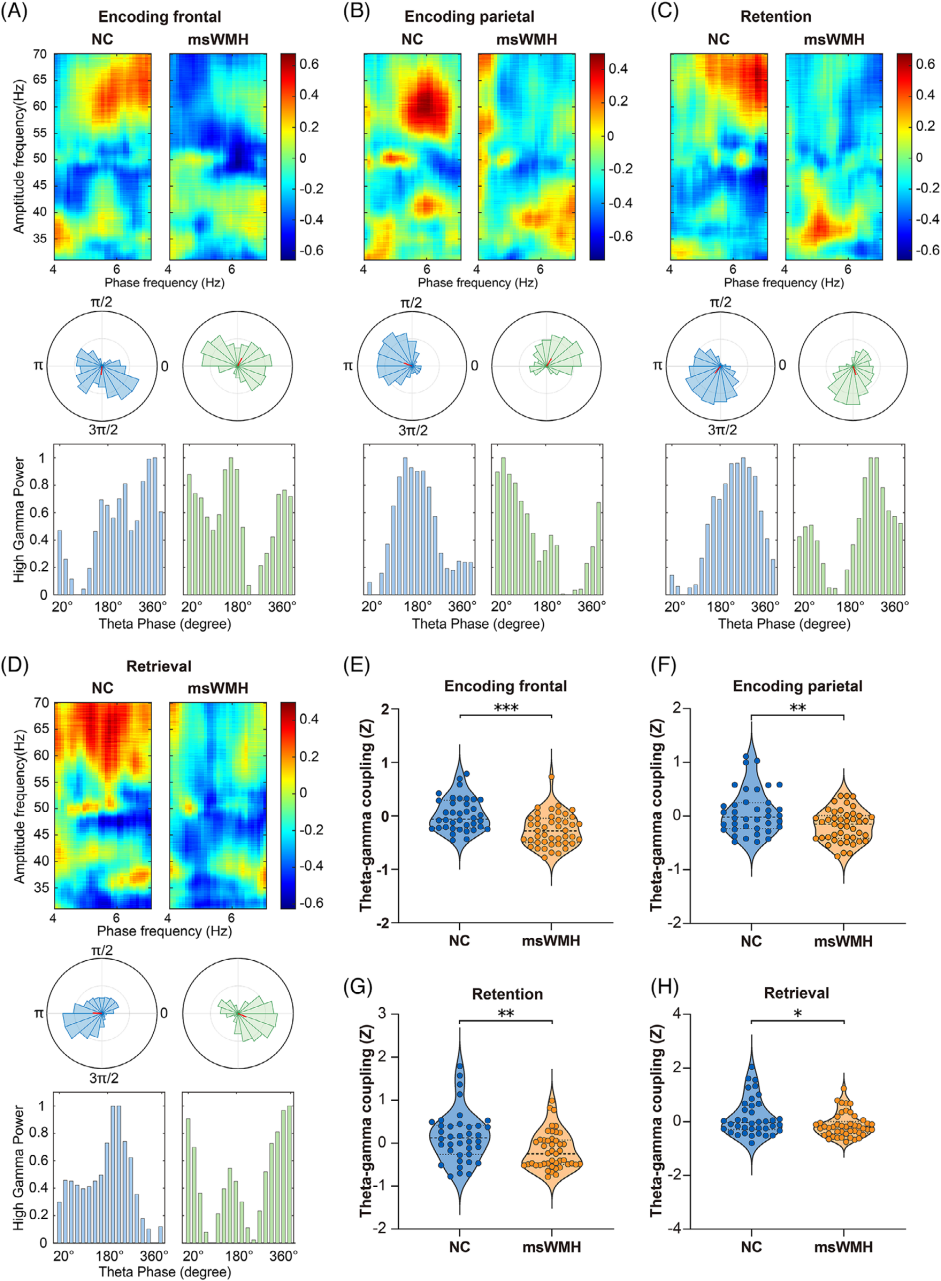
Theta‐gamma phase–amplitude coupling (PAC) during encoding, retention, and retrieval stages. (A,B) Averaged phase‐to‐amplitude comodulograms for the NCs and msWMH groups in the frontal and parietal region during the encoding stage. The corresponding polar distribution of normalized gamma amplitudes (z) across participants over theta phases within the significant PAC cluster identified during encoding. The thick red line indicates the mean direction (i.e., phase angle) of the mean resultant vector, and the corresponding histogram of gamma power across theta bins. (C,D) The averaged phase‐to‐amplitude comodulograms, polar distribution, and histogram of theta–gamma coupling for both groups during the retention and retrieval stages. (E,H) Group differences in theta–gamma coupling (z‐scores) between the NC and msWMH groups across encoding, retention, and retrieval stages. msWMH, moderate‐to‐severe white matter hyperintensity; NC, normal control. *** for *p* < 0.001, ** for *p* < 0.01, and * for *p* < 0.05.

The phase of theta oscillations modulates the amplitude of gamma activity to support information maintenance and integration in working memory.^18^, ^41^ Disrupted theta rhythms may impair this coupling. We therefore examined theta–gamma PAC during memory processing in patients with msWMHs. Compared with NCs, patients with msWMHs showed reduced theta–gamma PAC in the frontal (*t*_81_ = 4.338, *p* < 0.001; Figure 3A, E) and left parietal regions (*t*_81_ = 3.215, *p* = 0.002; Figure 3B, F) during encoding, in the left temporo‐parietal region (*t*_81_ = 2.931, *p *= 0.004; Figure 3C, G) during retention, and in the left frontal region (*t*_81_ = 2.397, *Pp* = 0.019; Figure 3D, H) during retrieval (see Table S3). These findings indicate that disruptions in theta oscillations and theta–gamma coupling may underlie face‐memory deficits in patients with msWMHs.

### 3.5 Association between electrophysiological metrics and cognitive performance

**FIGURE 4:**
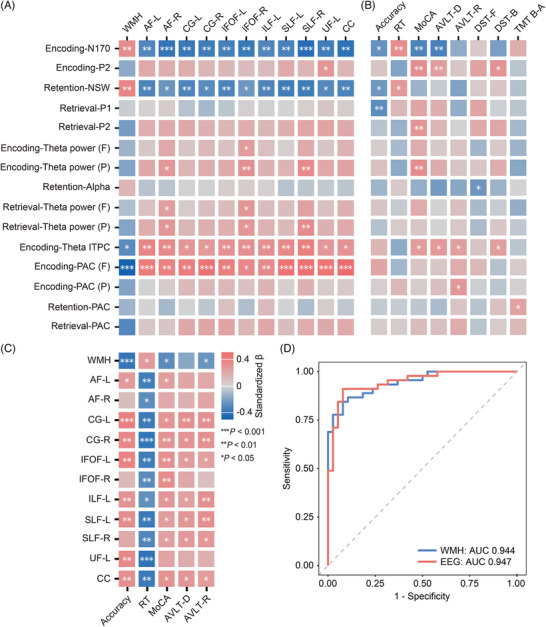
Associations among electrophysiological activities, WMH, white matter tract integrity, and neuropsychological scores, and their capacities in differentiating NC and msWMH groups. (A) Heatmap of associations between electrophysiological activities (ERPs, neural oscillations, and theta–gamma PAC), WMH load, and fractional anisotropy (FA) of white matter tracts in all participants. (B) Heatmap of associations between electrophysiological activities and neuropsychological scores in all participants. (C) Heatmap of associations between WMH load, FA of white matter tracts, and neuropsychological scores in all participants. (D) ROC curves demonstrating the diagnostic value of WMH load and EEG biomarkers (neural oscillations and theta–gamma PAC) in distinguishing NC and WMH groups. AF, arcuate fasciculus; AVLT‐D, Auditory Verbal Learning Test–delayed recall; AVLT‐R, Auditory Verbal Learning Test–recognition recall; CC, corpus callosum; CG, cingulum; DST‐F/B, Digit Span Test forward and backward; F, frontal; FX, fornix; IFOF, inferior fronto‐occipital fasciculus; ILF, inferior longitudinal fasciculus; ITPC, inter‐trial phase coherence; L, left; MoCA, Montreal Cognitive Assessment; P, parietal; PAC, phase–amplitude coupling; R, right; RT, reaction time; SLF, superior longitudinal fasciculus; TMT, Trail Making Test; UF, uncinate fasciculus; WMH, white matter hyperintensity. Blue indicates negative correlations; red indicates positive correlations. *** for *p *< 0.001, ** for *p* < 0.01, * for *p* < 0.05.

In the context of white matter progression, we examined potential associations among electrophysiological activity, behavioral performance, and neuropsychological measures. As shown in Figure 4B, after controlling for age, sex, and education, encoding‐related N170 (*β*_accuracy_ = −0.250, *p*_corrected_ = 0.021; *β*_RT_ = 0.314, *p*_corrected_ = 0.003) and retention‐related NSW (*β*_accuracy_ = −0.233, *p*_corrected_ = 0.030; *β*_RT_ = 0.260, *p*_corrected_ = 0.014) were significantly correlated with accuracy and RT in the face‐memory task. Moreover, encoding‐related N170 and P2 were significantly correlated with MoCA (N170: *β* = –0.314, *p*_corrected_ = 0.004; P2: *β* = 0.314, *p*_corrected_ = 0.004), AVLT‐delayed recall (N170: *β* = −0.323, *p*_corrected_ = 0.005; P2: *β* = 0.234, *p*_corrected_ = 0.044), and DST‐backward (P2: *β *= 0.291, *p*_corrected_ = 0.011). In addition, retrieval‐related P2 was correlated with MoCA (*β* = 0.282, *p*_corrected_ = 0.009) (see Table S4). Furthermore, behavioral performance was significantly associated with both memory function (AVLT‐immediate recall: *β* = 0.234, *p*_corrected_ = 0.041; AVLT‐recognition recall: *β* = 0.347, *p*_corrected_ = 0.003; DST‐backward: *β* = –0.298, *p*_corrected_ = 0.013) and global cognitive function (MoCA: *β* = −0.391, *p*_corrected_ < 0.001) (see Table S5). These results suggested that the aforementioned ERP metrics effectively and sensitively reflect memory‐related processing.

We further investigated the potential associations between neural oscillations and cognitive alterations. The results showed that reduced frontal theta power was associated with lower MoCA scores (*β* = 0.321, *p*_corrected_ = 0.003), and decreased theta phase synchronization was associated with declines in MoCA (*β *= 0.249, *p*_corrected_ = 0.023), AVLT‐delayed recall (*β *= 0.266, *p*_corrected_ = 0.019), AVLT‐recognition recall (*β *= 0.229, *p*_corrected_ = 0.045), and DST‐backward (*β *= 0.236, *p*_corrected_ = 0.037). Moreover, reduced encoding‐related left parietal theta–gamma PAC was associated with lower AVLT‐recognition recall (*β *= 0.271, *p*_corrected_ = 0.014) (see Table S4). These findings suggest that impairments in theta oscillation and theta–gamma PAC may serve as predictors of global cognitive function, particularly memory, in individuals with white matter damage.

### 3.6 Associations among WMHs, FA, and electrophysiological metrics

**FIGURE 5:**
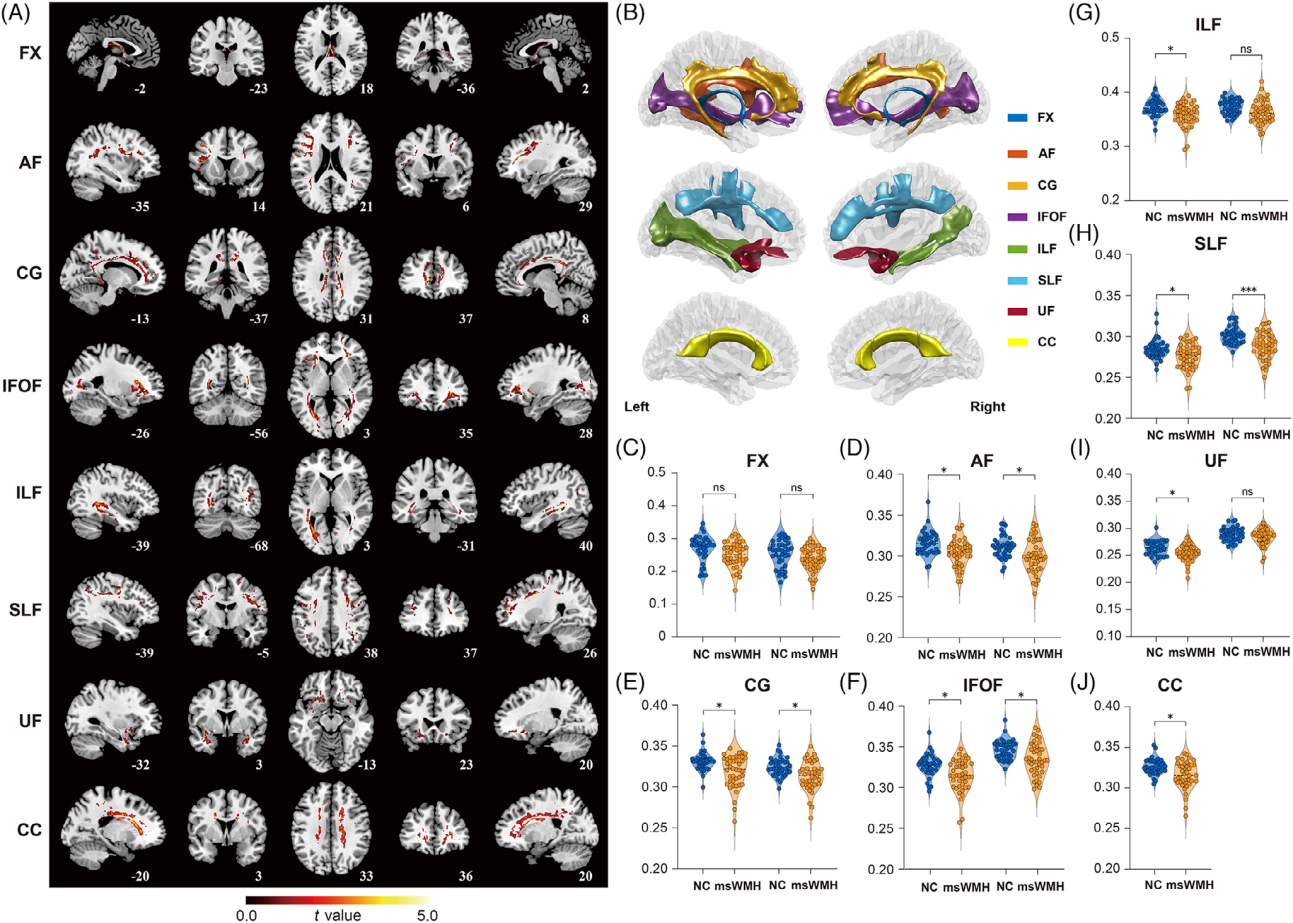
Altered fractional anisotropy (FA) of white matter tracts and tractography visualization in the NC and msWMH groups. (A) Comparison of FA in white matter tracts between NCs and msWMHs. (B) Sagittal view of investigated white matter tracts. (C–J) Group differences in FA values between NCs and msWMHs. AF, arcuate fasciculus; CC, corpus callosum; CG, cingulum; FX, fornix; IFOF, inferior fronto‐occipital fasciculus; ILF, inferior longitudinal fasciculus; SLF, superior longitudinal fasciculus; UF, uncinate fasciculus. Red areas indicate regions where FA was reduced in msWMHs compared to NCs. *** for *p* < 0.001, * for *p *< 0.05, and ns for not significant.

Patients with msWMHs exhibit not only increased WMH burden but also reduced white matter microstructural integrity. We further compared memory‐related white matter tract alterations between the NC and msWMH groups. The results showed that patients with msWMHs exhibited reduced FA values in almost all memory‐related white matter tracts, including the AF, CC, CG, IFOF, SLF, ILF, and UF (*ts* ≤ 4.332, *p*_corrected_ ≤ 0.030, FDR corrected; Figure 5A–J) (see Table S6 for details). Analyses of additional white matter tracts revealed some group differences, yet only limited associations with N170 amplitude and no cognitive effects (Tables S7–S9).

We further used linear regression models to examine the effects of white matter integrity on electrophysiological activities, adjusting for age, sex, and education. The results showed that encoding‐related P2 and N170 amplitudes, as well as retention‐related NSW amplitudes were associated with all memory‐related white matter tracts (*β* ≤ −0.236, *p*_corrected_ ≤ 0.038; Figure 4A). No correlation was revealed in the retrieval stage. In addition, encoding‐related frontal‐central theta power was associated with the integrity of the right IFOF (*β *= 0.249, *p*_corrected_ = 0.029; Figure 4A), and encoding‐related occipital theta power was associated with the integrity of the right AF, IFOF, and SLF (*β* ≥ 0.270, *p*_corrected_ ≤ 0.017; Figure 4A). Retrieval‐related frontal theta power was associated with the integrity of the right AF and IFOF (*β* ≥ 0.239, *p*_corrected_ ≤ 0.034; Figure 4A). Retrieval‐related left‐occipital theta power was associated with the integrity of the right AF, IFOF, and SLF (*β* ≥ 0.280, *p*_corrected_ ≤ 0.015; Figure 4A). Encoding‐related theta phase synchronization was associated with all memory‐related white matter tracts (*β* ≥ 0.256, *p*_corrected_ ≤ 0.026; Figure 4A). Furthermore, the encoding‐related frontal theta–gamma PAC was also associated with the integrity of all memory‐related white matter tracts (*β* ≥ 0.336, *p*_corrected_ ≤ 0.004; Figure 4A) (see Tables S10–S12 for details). These results suggest that the white matter microstructural integrity not only predicts the ERP activities, but also predicts the theta oscillation and theta–gamma coupling.

### 3.7 Electrophysiological metrics mediate the associations between white matter tracts and cognitive performance

We further examined the associations between white matter microstructural integrity and cognitive performance. Linear regression analyses revealed that almost all memory‐related white matter tract integrity was significantly associated with the accuracy and RT of face‐memory processing, as well as global cognition (MoCA) and memory performance (AVLT‐delayed recall, AVLT‐recognition) (*β* ≥ 0.233, *p*_corrected_ ≤ 0.047; Figure 4C), after adjusting for age, sex, and education (see Table S13 for details). These results support the notion that microstructural alterations of white matter can indeed influence global cognition and memory function.

**FIGURE 6:**
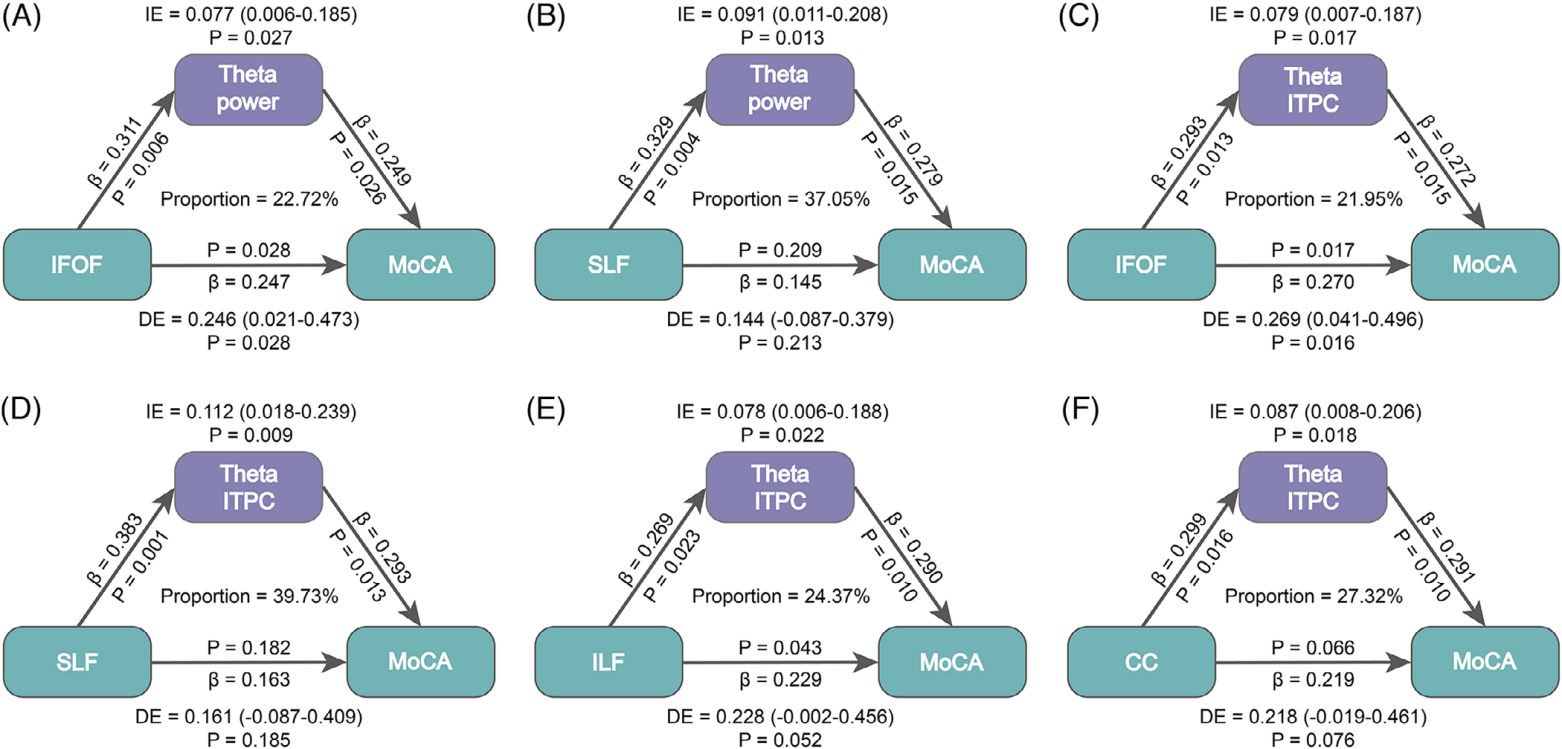
Mediation analysis results. (A,B) The inferior fronto‐occipital fasciculus (IFOF) and superior longitudinal fasciculus (SLF) influence global cognitive function through encoding‐related theta power. (C–F) The IFOF, SLF, inferior longitudinal fasciculus (ILF), and corpus callosum (CC) influence global cognitive function through encoding‐related theta phase synchronization.

Impaired white matter tracts may disrupt oscillatory communication between visual and memory networks, thereby contributing to deficits in memory and global cognition.^35^, ^42^ Based on this framework, we further tested the hypothesis that face‐memory–related neural oscillation mediates the relationship between white matter tract integrity and global cognition decline in the context of white matter progression. Mediation analyses showed that the integrity of right IFOF and SLF tracts impacts global cognition (MoCA) via encoding‐related occipital theta oscillations (all *p*s < 0.05; Figure 6A, B). Tract integrity represented by the IFOF, SLF, ILF, and CC affects global cognition (MoCA) through encoding‐related occipital theta phase synchronization (all *p*s < 0.05; Figure 6C–F) (see Table S14 for details). These results suggest that the memory‐encoding–related theta activities play a critical role in mediating the association between white matter tract (especially IFOF, SLF, and ILF) integrity and global cognition.

### 3.8 Electrophysiological metrics effectively distinguish NCs from msWMHs

Finally, we applied ROC analysis to further evaluate the capacity of face‐memory–related electrophysiological activity in distinguishing msWMHs and NCs. Electrophysiological metrics—including theta and alpha power, theta phase synchronization, and theta–gamma PAC across all three stages—effectively differentiated NCs from msWMHs, with an AUC of 0.947 (95% CI: 0.901–0.992; Figure 4D; see Table S15). This performance closely matched that obtained from WMH burden (AUC = 0.944, 95% CI: 0.899–0.988). These results indicate that electrophysiological activity related to face memory shows very high sensitivity in distinguishing NCs from msWMHs, suggesting that memory processing in msWMHs is markedly different from that in the NC group.

### 3.9 Associations between EPVS, lacunes, CMBs, and electrophysiological metrics

Other markers of CSVD were also assessed (see Supplementary Materials). Patients with msWMHs exhibited greater EPVS burden, whereas lacunes and CMBs did not differ between groups (Table S16). EPVS was closely related to WMH severity and emerged as the only CSVD marker associated with electrophysiological alterations (Table S17). After covariate adjustment, higher EPVS burden corresponded to reduced theta activity and theta ITPC during encoding, diminished theta–gamma coupling in frontal and temporo‐parietal regions, and lower P2 amplitude and occipital theta power during retrieval (Table S18). None of the CSVD indices showed associations with behavioral performance or cognition (Table S19).

## 4 DISCUSSION

This is the first study to integrate the OFDM task, EEG recordings, and DTI to investigate the neural mechanisms underlying memory processing impairments in patients with msWMHs. During the short‐term memory processing of faces, patients with msWMHs exhibited attenuated ERP activity across encoding, retention, and retrieval stages, suggesting a systemic deficit in memory processing. Alterations in theta power and phase synchronization during encoding and alpha power during retention may drive these memory deficits and further lead to abnormal theta–gamma PAC. Concurrently, tract‐based analyses revealed that msWMH individuals exhibited reduced microstructural integrity in several memory‐related white matter tracts, especially the IFOF, SLF, and ILF, compared with NCs. Mediation analyses further revealed that these tracts affect memory and global cognition via theta oscillatory activity. Finally, electrophysiological activities across encoding, retention, and retrieval stages effectively discriminated msWMH individuals from NCs, highlighting the potential clinical utility of OFDM task–based EEG measures.

### 4.1 Oscillatory neural dynamics underlying memory impairment in msWMH individuals

We systematically investigated, for the first time, the neural response patterns across different stages of memory processing in patients with msWMHs. As we expected, individuals with msWMHs showed both an altered N170 component and a reduced P2 component. The reduced N170 component reflects early impairment in the structural encoding of faces and has been linked to pathological cognitive decline.^43^ Abnormalities in the P2 component further reflect deficits in higher‐order configural processing and attentional resource allocation.^44^ Both of these results point to impaired face‐memory encoding capacity in individuals with msWMHs. Moreover, posterior NSW activity has been widely used as an index of memory maintenance during the retention stage,^21^, ^45^, ^46^ with larger memory loads eliciting greater NSW amplitudes.^13^ We demonstrated for the first time that individuals with msWMHs exhibit a significantly reduced NSW amplitude during the memory retention stage, confirming their impaired memory maintenance. The impairment in memory encoding and maintenance ultimately leads to abnormal ERP dynamics during the retrieval phase, manifested as reduced P1 and P2 components. Notably, although behavioral performance did not differ significantly between individuals with msWMHs and NCs, a declining trend was observed. These findings suggest that neural alterations may precede measurable behavioral deficits in the course of white matter pathology, underscoring the potential of electrophysiological markers of face memory as early indicators of cognitive decline. This implies that disruptions of white matter integrity may contribute to systematic memory processing deficits by interfering with specific neurophysiological activity patterns. In addition, preserved behavioral performance may reflect transient compensatory mechanisms during the progressive stage of WMH, with adaptive network reorganization and cortical plasticity supporting cognition via enhanced connectivity and alternative pathways.^47^, ^48^, ^49^ This interpretation aligns with brain resilience,^47^ suggesting that functional adaptability can temporarily offset structural disconnection and delay the manifestation of behavioral impairments.

Moreover, memory processing deficits in individuals with msWMHs are underpinned by abnormalities in theta and alpha activities. Successful memory encoding, retention, and retrieval rely on stable theta and alpha oscillations.^50^, ^51^, ^52^, ^53^ Enhanced theta power and phase synchronization both predict better memory performance.^17^, ^54^, ^55^ Reduced theta phase synchronization during memory activation in individuals with mild cognitive impairment (MCI) contributes to poorer working memory performance.^56^ In our study, not only did patients with msWMHs exhibit reduced theta power and phase synchronization, but these reductions were also linked to poorer memory and cognitive performance. Furthermore, alpha oscillations are thought to reflect sustained activation of the visual cortex to preserve memory representation. Altered alpha oscillations in patients with msWMHs indicated insufficient disinhibition and representation activation in the visual cortical area during memory maintenance. Of note, theta and alpha oscillations play a key role in shaping working‐memory capacity.^50^, ^57^ In particular, neuromodulation studies targeting theta oscillations, such as transcranial magnetic stimulation or theta‐frequency transcranial alternating current stimulation (tACS), can enhance working‐memory performance by upregulating theta‐band activity.^58^, ^59^ Likewise, phase‐locked alpha‐tACS applied during retention selectively changes alpha activity and working‐memory retention.^23^ These findings suggest that abnormal theta and alpha oscillations constitute a critical neurodynamic basis for reduced encoding precision and memory maintenance in msWMHs.

Critically, individuals with msWMHs exhibited reduced theta–gamma PAC during memory processing. The dynamic interaction between task‐related theta and gamma oscillations is considered a core mechanism for integrating information across temporal scales. Theta rhythms provide a phase reference for gamma oscillations, thereby temporally organizing discrete information into stable memory traces for subsequent encoding and retrieval.^60^ Prior studies have shown that theta–gamma PAC predicts the accuracy of memory performance; of note, stronger coupling reflects more efficient encoding and retrieval, leading to superior memory outcomes.^61^, ^62^ In populations with MCI and Alzheimer's disease (AD), reduced theta–gamma PAC has been significantly associated with working memory deficits.^63^ Consistent with these findings, our study demonstrated that reduced theta–gamma PAC during memory processing was associated with subsequent cognitive decline, suggesting that coupling deficits may reflect disrupted multiscale neural coding mechanisms in msWMH individuals. These results suggest that white matter tract damage weakens theta phase stability, thereby reducing the effective modulation of local gamma activity, which in turn diminishes coupling strength and ultimately disrupts the temporally precise integration of neural information.

### 4.2 White matter tract alterations and their associations with EEG metrics in msWMHs

Previous studies have focused primarily on macrostructural white matter changes related to memory processing,^64^, ^65^ but microstructural alterations remain underexplored. In this study, we further demonstrated white matter microstructural impairments associated with memory processing in silent msWMH individuals. Compared with healthy older adults, these patients exhibited reduced integrity in key tracts, particularly the bilateral IFOF, SLF, and UF, and the left ILF. These alterations have also been reported in WMH individuals with overt cognitive impairment.^66^, ^67^ These results suggest that memory‐related tracts may already be selectively damaged in the silent stage of the disease. Moreover, the integrity of the white matter tracts was significantly associated with altered N170 activity during memory encoding and altered NSW activity during memory maintenance. These results further suggest that memory‐related white matter microstructural damage affects not only face encoding but also memory maintenance.

### 4.3 Theta oscillations mediate the effects of white matter tracts on cognition

Moreover, the widespread white matter damage, especially in tracts linking occipital, temporal, and frontal regions whose microstructural integrity is consistently implicated in cognitive decline^67^, ^68^ was closely associated with reduced memory‐processing–related theta activity, particularly diminished theta phase synchronization. This structural disconnection likely disrupts theta synchronization and reduces the efficiency of memory‐related information transfer, thereby providing a mechanistic pathway to cognitive decline. A prevailing view is that vascular factors, such as hypoperfusion and chronic hypoxia,^69^ blood–brain barrier disruption,^70^ and endothelial dysfunction,^71^ damage myelin structures in the white matter and impair repair mechanisms. These changes interfere with the transmission of neural oscillations across neural circuits, ultimately compromising memory and cognitive functions.^35^, ^72^ In addition, impaired neurovascular coupling may also contribute to electrophysiological changes,^73^ and provide a complementary account for the structure–function relationships in msWMHs. Here, we demonstrate for the first time that memory‐related tracts—the IFOF, SLF, ILF, and CC—influence cognitive function through theta oscillations. The dynamic interplay between structure and function reveals a covariation pattern between white matter microstructure and theta activity during memory processing.

### 4.4 Neural oscillatory activity reliably differentiates NC from msWMH individuals

Finally, ROC analysis revealed that neural oscillatory activity and WMH burden showed comparable performance in distinguishing msWMH individuals from healthy older adults. The reliable differentiating ability of face‐memory–related neural oscillatory activity suggests that the memory processing in msWMHs differs markedly from that in NCs, and also suggests that neural oscillations can indeed sensitively reflect the impact of WMH on brain function. The comparable discriminative power also implies a tight correspondence between structural damage and functional disturbance, indirectly corroborating the mechanism by which WMHs disrupt information transmission and network synchronization to produce cognitive impairment. Moreover, given the noninvasive and portable nature of EEG, it may serve as a promising functional biomarker for detecting and predicting cognitive decline in CSVD. Compared with imaging‐based “static burden” such as WMH burden, neural oscillations reflect task‐related dynamic network states, which may lie closer to the actual mechanisms of cognitive impairment. Further research is necessary to examine the potential of EEG biomarkers in predicting cognitive decline associated with white matter degeneration.

This study has some limitations. First, due to its cross‐sectional design, this study limits the ability to infer causal relationships between task‐related EEG alterations and cognitive decline. Longitudinal investigations are therefore needed to clarify the temporal dynamics and underlying mechanisms. Second, the present study revealed only alterations in local neural oscillatory activity; future research should further investigate EEG‐based brain network changes and their potential associations with white matter structure. In addition, the potential relationship between neural oscillation and neurovascular coupling remains to be further elucidated. Finally, as our data were derived from a single‐center WMH outpatient clinic, these findings should be generalized with caution. Replication across diverse populations and multiple centers is warranted to mitigate potential selection bias.

In summary, using the OFDM task, we found that msWMH patients exhibited hidden neural deficits in VSTM processing, characterized by altered theta and alpha oscillations and disrupted theta–gamma PAC across different memory stages. Moreover, within the context of white matter pathology, theta activity mediated the effects of the IFOF, SLF, ILF, and UF on global cognitive functions. Finally, electrophysiological activities derived from encoding, retention, and retrieval stages effectively distinguished NCs from individuals with msWMH, highlighting their potential utility for early screening and diagnosis in this population. Such EEG‐based functional markers may provide sensitive tools for identifying individuals at risk and monitoring the progression of WMHs.

## AUTHOR CONTRIBUTIONS

Aonan Li and Lei Liu drafted the manuscript and prepared the tables/figures. Yi Xing and Yi Tang designed and supervised the work. Aonan Li and Yuanyuan Lu collected the samples and data. Aonan Li, Lei Liu, and Yueyan Bian analyzed the data. Aonan Li, Lei Liu, Xiuqin Jia, and Yuanyuan Chen revised the manuscript critically. All authors read and approved the final manuscript.

## CONFLICT OF INTEREST STATEMENT

The authors declare that they have no competing interests. Any author disclosures are available in the Supporting Information.

## CONSENT STATEMENT

This study was approved by the Institutional Review Board of Xuanwu Hospital, Capital Medical University (No. 2024092), in accordance with the 1964 Declaration of Helsinki and its later amendments or comparable ethical standards. Written informed consent was obtained from all participants.

## Supporting information



Supporting Information

Supporting Information

## Data Availability

The datasets analyzed during the current study are not publicly available due to patient privacy purposes, but are available from the corresponding author on reasonable request.
