# Supplementary material for: Silent yet impaired: Hidden memory processing deficits in asymptomatic individuals with moderate‐to‐severe white matter hyperintensities
Source: Alzheimers Dement. 2026 Jan 7;22(1):e71084. doi: 10.1002/alz.71084 (PMC12778417; doi:10.1002/alz.71084)
Supplement: Supplementary file 1 — Supporting Information [file ALZ-22-e71084-s001.docx]

**Supplementary**

**Supplementary Methods**

**Inclusion and exclusion criteria for patients with moderate-to-severe white matter hyperintensities.**

**Inclusion criteria：**

1. Age 55-80 years;

2. The presence of cerebral small vessel disease markers on magnetic resonance imaging shows any of the following:

(1) Moderate to severe white matter hyperintensity (WMH) changes in the subcortical or periventricular regions, detected on T2-weighted (T2WI) or fluid-attenuated inversion recovery (FLAIR) MRI sequences, corresponding to Fazekas grade 2-3;

(2) No or mild hippocampal/entorhinal cortex atrophy, medial temporal lobe atrophy scale (MTA) score of 0 or 1;

3. Neurological examination revealed no obvious clinical signs or positive findings;

4. Signing of informed consent.

**Exclusion criteria:**

1. Acute ischemic infarction with a maximum diameter of more than 20mm on diffusion weighted imaging;

2. Acute hemorrhagic stroke;

3. Acute subarachnoid hemorrhage, untreated cerebrovascular malformation, or untreated hemangioma with a diameter of more than 3mm;

4. Definite diagnosis of neurodegenerative diseases, such as Alzheimer’s disease and Parkinson’s disease;

5. Definite diagnosis of WMH of presumed non-vascular origin, such as multiple sclerosis, adult-onset leukodystrophy, and metabolic encephalopathy;

6. Definite diagnosis of mental disorders according to the 5th edition of the Diagnostic and Statistical Manual of Mental Disorders (DSM-V) criteria;

7. Contradictions of magnetic resonance imaging examination, e.g., claustrophobia;

8. Severe organic diseases, such as malignant tumors, with a life expectancy of less than 5 years;

9. Participant in other clinical trials at the same time.

Additional Analyses of CSVD markers and EEG measures

Enlarged perivascular spaces measurement

Whole-brain EPVS burden was quantified using a multimodal deep learning segmentation framework trained and validated on T1- and T2-weighted MRI in cohorts with cerebral small vessel disease.^1,2^ This model has demonstrated high reliability in capturing EPVS-related features across diverse imaging conditions. The network produced voxel-wise probability maps for each participant, from which subject-specific EPVS masks were derived using a probability threshold of 0.1—an empirically optimized cutoff that balanced sensitivity with false-positive control and was further corroborated by our visual inspection. These binarized maps were used to compute total EPVS volume, which was subsequently normalized to total intracranial volume and multiplied by 1000 to account for individual variability in brain size.

**Results**

The results showed that patients with msWMH exhibited enlarged PVS (t = –7.004, *P* < 0.001), whereas lacunes and CMBs did not differ significantly between groups (Table S16). Prior studies have reported co-occurrence and interrelationships among WMH and EPVS.^3,4^ Consistent with these findings, EPVS (ρ = 0.648, *P* < 0.001) was positively correlated with WMH burden (Table S17).

We further applied linear regression models to examine the effects of these CSVD markers on electrophysiological activity, adjusting for age, sex, and education. The results showed that EPVS was associated with fronto-central theta power (β = -0.236, *P*_corrected_ = 0.033; Table S18) and occipital theta ITPC (β = -0.234, *P*_corrected_ = 0.035; Table S18) during encoding, as well as frontal theta–gamma PAC (β = -0.429, *P*_corrected_ < 0.001; Table S18). During the retention period, EPVS was associated with left temporal parietal theta–gamma PAC (β = -0.231, *P*_corrected_ = 0.046; Table S18). During retrieval, EPVS was associated with P2 amplitude (β = -0.300, *P*_corrected_ = 0.007; Table S18) and left occipital theta power (β = -0.227, *P*_corrected_ = 0.042; Table S18). Finally, no significant associations were observed between lacunes, EPVS, CMBs, and behavioral performance or cognitive (Table S19).

# **
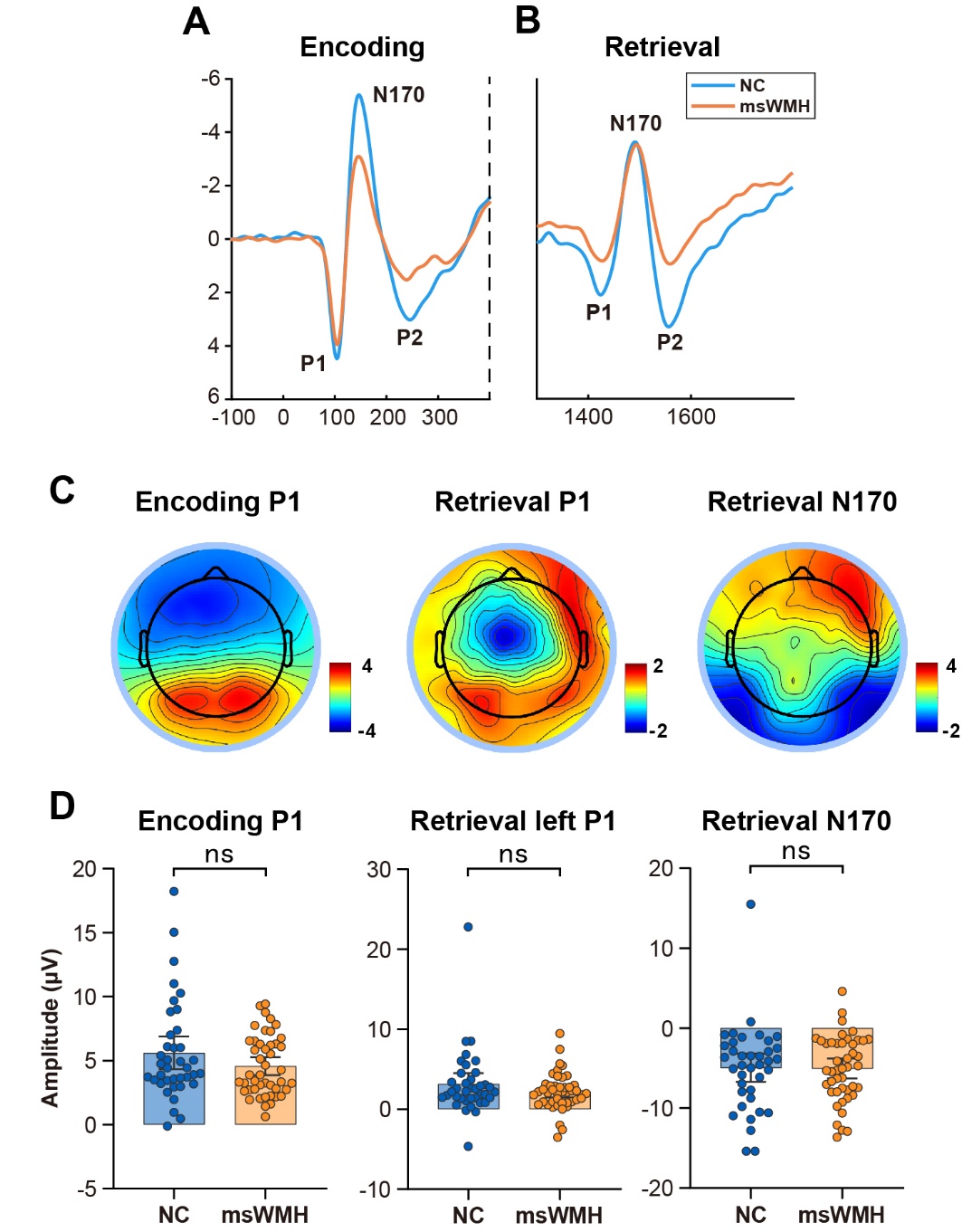
Figure S1. Behavioral and ERP results during the occluded face delayed matching (OFDM) task in NC and msWMH individuals.**

(A) Posterior P1 components evoked by occluded faces during the encoding stage in both groups. (B) Left occipital P1 and right temporo-occipital N170 components were evoked by intact faces during the retrieval stage in both groups. (C) Topographical maps of encoding-related P1 and retrieval-related P1 and N170 components. (G) Bar charts of the three ERP components in the two groups. ns, not significant; NC, normal control; WMH, white matter hyperintensities.


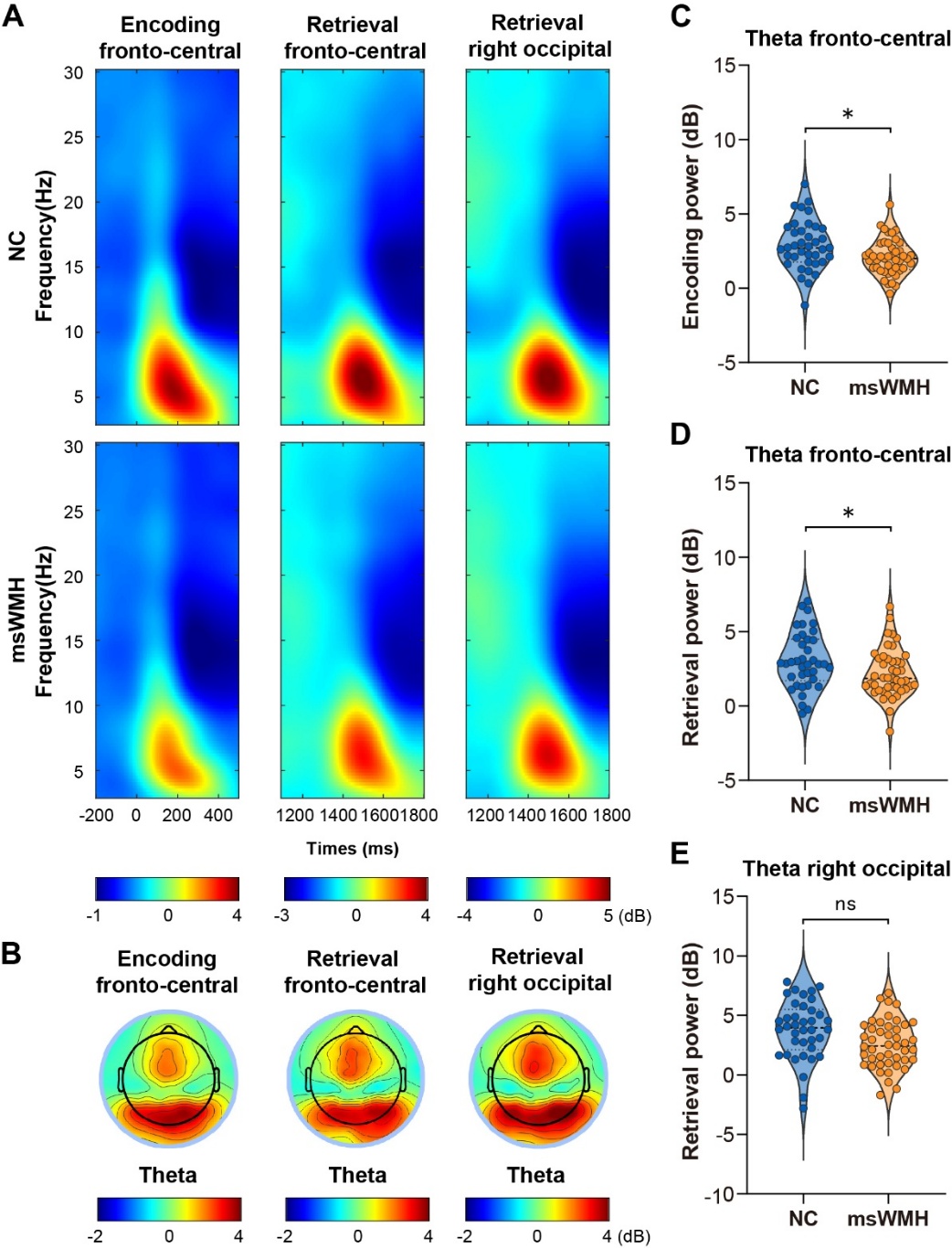
Figure S2. Comparisons of power between the two groups during the encoding and retrieval stages.

(A) Grand-average event-related spectral perturbations (ERSPs) in both groups during the encoding and retrieval stages. Theta power was clearly altered in the frontal region during the encoding and retrieval stages, whereas no significant differences in theta power were observed in the right occipital region during the retrieval stage. (B) Grand-averaged topographical distributions of theta band power during the encoding and retrieval stages. (C–E) Bar charts showing group differences in theta band power in the frontal region during the encoding and retrieval stages, and in the right occipital region during the retrieval stage.

Table S1. Differences between NC and msWMH in event-related potentials.

|  | **NC (n = 38)** | **msWMH (n = 45)** | **t** | ***P*** |
| --- | --- | --- | --- | --- |
| **Encoding** |  |  |  |  |
| P1 | 5.599 (3.894) | 4.574 (2.35) | 1.477 | 0.144 |
| N170 | -5.919 (3.800) | -3.695 (3.023) | -2.968 | **0.004** |
| P2 | 4.493 (2.768) | 3.239 (2.255) | 2.276 | **0.026** |
| **Retention** |  |  |  |  |
| NSW | -4.966 (5.372) | -5.054 (4.113) | -2.781 | **0.007** |
| **Retrieval** |  |  |  |  |
| P1 (left occipital) | 3.131 (4.108) | 2.170 (2.315) | 1.339 | 0.184 |
| P1 (right occipital) | 2.715 (3.219) | 1.568 (1.836) | 2.031 | **0.046** |
| N170 | -2.235 (1.463) | -1.179 (1.916) | 0.084 | 0.933 |
| P2 | 4.223 (3.829) | 1.903 (2.590) | 3.275 | **0.002** |

Continuous data are presented as mean (standard deviation). Abbreviations: NSW, negative slow wave. Bold type indicates statistical significance (*P* < 0.05).

Table S2. Differences between NC and msWMH in neural oscillation.

|  | **NC (n = 38)** | **msWMH** **(n = 45)** | **t** | ***P*** |
| --- | --- | --- | --- | --- |
| **ERSP** |  |  |  |  |
| ***Encoding*** |  |  |  |  |
| Theta (fronto-central) | 2.898 (1.655) | 2.074 (1.200) | 2.623 | **0.010** |
| Theta (occipital) | 4.155 (1.900) | 2.931 (1.618) | 3.168 | **0.002** |
| ***Retention*** |  |  |  |  |
| Alpha (right occipital) | -0.931 (1.682) | -0.159 (1.417) | -2.270 | **0.026** |
| ***Retrieval*** |  |  |  |  |
| Theta (fronto-central) | 3.060 (1.902) | 2.200 (1.623) | 2.198 | **0.031** |
| Theta (left occipital) | 3.798 (2.454) | 2.570 (1.991) | 2.516 | **0.014** |
| Theta (right occipital) | 4.280 (2.648) | 3.520 (2.283) | 1.408 | 0.163 |
| **ITPC** |  |  |  |  |
| ***Encoding*** |  |  |  |  |
| Theta (occipital) | 0.654 (0.135) | 0.556 (0.134) | 3.297 | **0.001** |
| ***Retrieval*** |  |  |  |  |
| Theta (right occipital) | 0.644 (0.130) | 0.591 (0.151) | 0.937 | 0.352 |

Continuous data are presented as mean (standard deviation). Abbreviations: ERSP, event-related spectral perturbation; ITPC, inter-trial phase coherence. Bold type indicates statistical significance (*P* < 0.05).

Table S3. Differences between NC and msWMH in theta-gamma PAC.

|  | **NC (n = 38)** | **msWMH (n = 45)** | **t** | ***P*** |
| --- | --- | --- | --- | --- |
| **Encoding** |  |  |  |  |
| Frontal | 0.026 (0.307) | -0.265 (0.301) | 4.338 | **< 0.001** |
| Left parietal | 0.069 (0.415) | -0.183 (0.295) | 3.215 | **0.002** |
| **Retention** |  |  |  |  |
| Left temporo-parietal | 0.158 (0.598) | -0.173 (0.429) | 2.931 | **0.004** |
| **Retrieval** |  |  |  |  |
| Left frontal | 0.169 (0.689) | -0.132 (0.444) | 2.397 | **0.019** |

Continuous data are presented as mean (standard deviation). Electrodes for each region were selected as follows: frontal (averaged across E11, E13, E124) and left parietal (averaged across E42, E47, E52) for encoding, left temporo-parietal (E50) for retention, and left frontal (E18) for retrieval. Abbreviation: PAC, phase-amplitude coupling. Bold type indicates statistical significance (*P* < 0.05).

Table S4. Association between electrophysiological metrics and cognitive performance.

|  | **Accuracy** | | **RT** | | **MoCA** | | **AVLT-D** | | **AVLT-R** | | **DST-F** | | **DST-B** | | **TMTB-A** | |
| --- | --- | --- | --- | --- | --- | --- | --- | --- | --- | --- | --- | --- | --- | --- | --- | --- |
|  | **β** | ***P*** | **β** | ***P*** | **β** | ***P*** | **β** | ***P*** | **β** | ***P*** | **β** | ***P*** | **β** | ***P*** | **β** | ***P*** |
| **ERP** |  |  |  |  |  |  |  |  |  |  |  |  |  |  |  |  |
| ***Encoding*** |  |  |  |  |  |  |  |  |  |  |  |  |  |  |  |  |
| N170 | -0.250 | **0.021** | 0.314 | **0.003** | -0.314 | **0.004** | -0.323 | **0.005** | -0.154 | 0.188 | 0.047 | 0.685 | -0.205 | 0.077 | -0.059 | 0.612 |
| P2 | 0.062 | 0.576 | -0.185 | 0.086 | 0.314 | **0.004** | 0.234 | **0.044** | 0.050 | 0.668 | 0.222 | 0.052 | 0.291 | **0.011** | 0.168 | 0.146 |
| ***Retention*** |  |  |  |  |  |  |  |  |  |  |  |  |  |  |  |  |
| NSW | -0.233 | **0.030** | 0.260 | **0.014** | -0.125 | 0.261 | -0.034 | 0.772 | -0.189 | 0.101 | 0.035 | 0.757 | 0.058 | 0.615 | -0.071 | 0.539 |
| ***Retrieval*** |  |  |  |  |  |  |  |  |  |  |  |  |  |  |  |  |
| P1 right | -0.275 | **0.008** | 0.121 | 0.247 | 0.206 | 0.056 | 0.168 | 0.137 | -0.146 | 0.194 | 0.209 | 0.058 | -0.014 | 0.898 | -0.056 | 0.619 |
| P2 | -0.057 | 0.599 | -0.130 | 0.221 | 0.282 | **0.009** | 0.132 | 0.251 | 0.001 | 0.992 | 0.181 | 0.107 | 0.197 | 0.082 | -0.106 | 0.353 |
| **ERSP** |  |  |  |  |  |  |  |  |  |  |  |  |  |  |  |  |
| ***Encoding*** |  |  |  |  |  |  |  |  |  |  |  |  |  |  |  |  |
| Theta (fronto-central) | 0.029 | 0.793 | -0.097 | 0.370 | 0.321 | **0.003** | 0.170 | 0.143 | 0.067 | 0.563 | 0.050 | 0.664 | 0.164 | 0.155 | -0.172 | 0.133 |
| Theta (occipital) | 0.076 | 0.484 | -0.156 | 0.146 | 0.173 | 0.121 | 0.041 | 0.727 | 0.073 | 0.534 | -0.115 | 0.316 | 0.042 | 0.719 | -0.125 | 0.277 |
| ***Retention*** |  |  |  |  |  |  |  |  |  |  |  |  |  |  |  |  |
| Alpha (right occipital) | -0.120 | 0.281 | 0.048 | 0.661 | -0.139 | 0.222 | -0.219 | 0.063 | -0.168 | 0.154 | -0.234 | 0.043 | -0.123 | 0.295 | -0.002 | 0.984 |
| ***Retrieval*** |  |  |  |  |  |  |  |  |  |  |  |  |  |  |  |  |
| Theta (fronto-central) | 0.064 | 0.562 | -0.035 | 0.746 | 0.171 | 0.126 | 0.008 | 0.945 | 0.086 | 0.465 | -0.096 | 0.404 | -0.029 | 0.806 | -0.143 | 0.216 |
| Theta (left occipital) | -0.069 | 0.524 | 0.039 | 0.712 | 0.052 | 0.641 | 0.006 | 0.960 | 0.048 | 0.678 | -0.091 | 0.422 | 0.051 | 0.659 | -0.046 | 0.687 |
| **ITPC** |  |  |  |  |  |  |  |  |  |  |  |  |  |  |  |  |
| ***Encoding*** |  |  |  |  |  |  |  |  |  |  |  |  |  |  |  |  |
| Theta (occipital) | 0.132 | 0.219 | -0.158 | 0.137 | 0.249 | **0.023** | 0.266 | **0.019** | 0.229 | **0.045** | 0.066 | 0.558 | 0.236 | **0.037** | -0.054 | 0.638 |
| **PAC** |  |  |  |  |  |  |  |  |  |  |  |  |  |  |  |  |
| ***Encoding*** |  |  |  |  |  |  |  |  |  |  |  |  |  |  |  |  |
| Frontal | 0.203 | 0.050 | -0.111 | 0.281 | 0.066 | 0.539 | 0.057 | 0.612 | 0.176 | 0.113 | 0.116 | 0.288 | -0.070 | 0.526 | 0.036 | 0.746 |
| Left parietal | 0.020 | 0.850 | 0.121 | 0.240 | 0.026 | 0.812 | 0.086 | 0.444 | 0.271 | **0.014** | -0.017 | 0.874 | -0.054 | 0.628 | 0.017 | 0.877 |
| ***Retention*** |  |  |  |  |  |  |  |  |  |  |  |  |  |  |  |  |
| Left tempo-parietal | -0.037 | 0.724 | -0.033 | 0.747 | -0.049 | 0.647 | -0.093 | 0.403 | 0.069 | 0.533 | -0.061 | 0.577 | 0.079 | 0.476 | 0.221 | 0.043 |
| ***Retrieval*** |  |  |  |  |  |  |  |  |  |  |  |  |  |  |  |  |
| Left frontal | 0.025 | 0.814 | 0.033 | 0.750 | 0.101 | 0.346 | 0.089 | 0.424 | 0.175 | 0.114 | -0.063 | 0.564 | 0.037 | 0.738 | 0.114 | 0.302 |

Abbreviations: ERP, event-related potential; ERSP, event-related spectral perturbation; ITPC, inter-trial phase coherence; PAC, phase-amplitude coupling; RT, reaction time; MoCA, Montreal Cognitive Assessment; AVLT-D, Auditory Verbal Learning Test delayed recall; AVLT-R, Auditory Verbal Learning Test recognition recall; DST-F/B, Digit Span Test forward and backward; TMT, Trail Making Test. Analyses were adjusted for age, sex, and education. Bold type indicates statistical significance (*P* < 0.05).

Table S5. Association between OFDM task behavioral measures and cognitive performance.

|  | **Accuracy** | | **Reaction Time** | |
| --- | --- | --- | --- | --- |
|  | **β** | ***P*** | **β** | ***P*** |
| MoCA | 0.432 | 0.995 | -0.391 | **<0.001** |
| AVLT-I | 0.234 | **0.041** | -0.030 | 0.801 |
| AVLT-D | 0.177 | 0.144 | -0.124 | 0.310 |
| AVLT-R | 0.347 | **0.003** | -0.086 | 0.482 |
| DST F | 0.127 | 0.284 | -0.081 | 0.499 |
| DST B | 0.184 | 0.124 | -0.298 | **0.013** |
| TMT B-A | -0.130 | 0.278 | 0.067 | 0.579 |

Abbreviations: OFDM, Occluded Face Delayed Matching; RT, reaction time; MoCA, Montreal Cognitive Assessment; AVLT-I, Auditory Verbal Learning Test immediate recall; AVLT-D, Auditory Verbal Learning Test delayed recall; AVLT-R, Auditory Verbal Learning Test recognition recall; DST-F/B, Digit Span Test forward and backward; TMT, Trail Making Test. Analyses were adjusted for age, sex, and education. Bold type indicates statistical significance (*P* < 0.05).

Table S6. Differences in the white matter tracts integrity between NC and msWMH.

|  | **NC (n = 38)** | **msWMH (n = 45)** | **t** | ***P* value** | ***P* adjusted** |
| --- | --- | --- | --- | --- | --- |
| FX-L | 0.269 (0.040) | 0.250 (0.038) | 2.148 | **0.035** | 0.105 |
| FX-R | 0.254 (0.040) | 0.237 (0.035) | 2.010 | **0.048** | 0.096 |
| AF-L | 0.316 (0.015) | 0.304 (0.016) | 3.535 | **0.001** | **0.014** |
| AF-R | 0.311 (0.014) | 0.300 (0.020) | 3.016 | **0.003** | **0.021** |
| CG-L | 0.331 (0.011) | 0.319 (0.020) | 3.213 | **0.002** | **0.018** |
| CG-R | 0.324 (0.011) | 0.313 (0.018) | 3.177 | **0.002** | **0.016** |
| IFOF-L | 0.327 (0.014) | 0.314 (0.019) | 3.390 | **0.001** | **0.013** |
| IFOF-R | 0.346 (0.012) | 0.334 (0.020) | 3.344 | **0.001** | **0.012** |
| ILF-L | 0.373 (0.016) | 0.358 (0.021) | 3.613 | **0.001** | **0.011** |
| ILF-R | 0.373 (0.013) | 0.364 (0.021) | 2.381 | **0.020** | 0.080 |
| SLF-L | 0.285 (0.012) | 0.276 (0.015) | 2.874 | **0.005** | **0.030** |
| SLF-R | 0.302 (0.011) | 0.288 (0.016) | 4.332 | **<0.001** | **<0.001** |
| UF-L | 0.264 (0.013) | 0.255 (0.015) | 2.851 | **0.006** | **0.030** |
| UF-R | 0.289 (0.013) | 0.284 (0.015) | 1.617 | **0.110** | 0.110 |
| CC | 0.326 (0.010) | 0.315 (0.017) | 3.438 | **0.001** | **0.010** |

Continuous data are presented as mean (standard deviation). Abbreviations: FX, fornix; AF, arcuate fasciculus; CG, cingulum; IFOF, inferior fronto-occipital fasciculus; ILF, inferior longitudinal fasciculus; SLF, superior longitudinal fasciculus; UF, uncinate fasciculus; CC, corpus callosum. L, left; R, right. P values are FDR-adjusted; bold indicates significance (*P* < 0.05).

Table S7. Differences in the additional white matter tracts integrity between NC and msWMH.

|  | **NC**  **(n = 38)** | **msWMH**  **(n = 45)** | **t** | ***P* value** | ***P* adjusted** |
| --- | --- | --- | --- | --- | --- |
| AR-L | 0.494 (0.025) | 0.237 (0.035) | 1.223 | 0.225 | 0.225 |
| AR-R | 0.487 (0.019) | 0.250 (0.038) | 2.894 | 0.005 | **0.035** |
| OR-L | 0.412 (0.021) | 0.397 (0.025) | 2.949 | 0.004 | **0.040** |
| OR-R | 0.436 (0.018) | 0.419 (0.031) | 2.845 | 0.006 | **0.030** |
| FAT-L | 0.294 (0.015) | 0.411 (0.020) | 2.602 | 0.011 | **0.044** |
| FAT-R | 0.306 (0.015) | 0.412 (0.022) | 2.141 | 0.035 | 0.105 |
| Cerebellum-L | 0.177 (0.010) | 0.171(0.010) | 2.834 | 0.006 | **0.036** |
| Cerebellum-R | 0.180 (0.011) | 0.175 (0.008) | 2.124 | 0.037 | 0.074 |
| MLF-L | 0.508 (0.028) | 0.494 (0.404) | 2.954 | 0.004 | **0.036** |
| MLF-R | 0.455 (0.023) | 0.438 (0.028) | 2.994 | 0.004 | **0.032** |

Continuous data are presented as mean (standard deviation). Abbreviations: AR, acoustic radiation; OR, optic radiation; FAT, Frontal aslant tract; MLF, medial longitudinal fasciculus; L, left; R, right. P values are FDR-adjusted; Bold indicates significance (*P* < 0.05).

Table S8. Associations between the additional white matter tracts integrity and ERP metrics.

|  | **Encoding** | | | | **Retention** | | | | **Retrieval** | |
| --- | --- | --- | --- | --- | --- | --- | --- | --- | --- | --- |
|  | **N170** | | **P2** | | **NSW** | | **P1** | | **N170** | |
|  | **β** | **P** | **β** | **P** | **β** | **P** | **β** | **P** | **β** | **P** |
| AR-R | -0.245 | 0.026 | 0.056 | 0.623 | -0.126 | 0.259 | 0.102 | 0.384 | -0.038 | 0.731 |
| OR-L | -0.281 | **0.010** | 0.069 | 0.538 | -0.203 | 0.057 | 0.026 | 0.823 | -0.001 | 0.990 |
| OR-R | -0.299 | **0.006** | 0.071 | 0.530 | -0.216 | 0.052 | 0.006 | 0.962 | -0.041 | 0.710 |
| FAT-L | -0.272 | **0.017** | 0.202 | 0.082 | -0.156 | 0.179 | 0.154 | 0.207 | -0.003 | 0.979 |
| Cerebellum-L | -0.178 | 0.155 | 0.062 | 0.623 | -0.023 | 0.858 | -0.056 | 0.673 | 0.026 | 0.836 |
| MLF-L | -0.071 | 0.535 | -0.006 | 0.960 | -0.101 | 0.379 | -0.018 | 0.879 | -0.052 | 0.647 |
| MLF-R | -0.191 | 0.082 | 0.202 | 0.069 | -0.173 | 0.120 | 0.035 | 0.766 | -0.008 | 0.942 |

Abbreviations: ERP, event-related potential; AR, acoustic radiation; OR, optic radiation; FAT, Frontal aslant tract; MLF, medial longitudinal fasciculus; L, left; R, right. Analyses were adjusted for age, sex, and education. Bold type indicates statistical significance (*P* < 0.05).

Table S9. Associations between the additional white matter tracts integrity and cognitive.

|  | **Accuracy** | | **AVLT-I** | | **AVLT-D** | | **AVLT-R** | |
| --- | --- | --- | --- | --- | --- | --- | --- | --- |
|  | **β** | **P** | **β** | **P** | **β** | **P** | **β** | **P** |
| AR-R | -0.034 | 0.755 | -0.160 | 0.149 | 0.051 | 0.662 | -0.019 | 0.871 |
| OR-L | 0.169 | 0.118 | 0.056 | 0.616 | 0.196 | 0.086 | 0.207 | 0.053 |
| OR-R | 0.167 | 0.127 | -0.020 | 0.858 | 0.033 | 0.774 | 0.133 | 0.244 |
| FAT-L | 0.169 | 0.138 | 0.030 | 0.798 | 0.221 | 0.064 | 0.153 | 0.197 |
| Cerebellum-L | 0.154 | 0.209 | 0.085 | 0.498 | 0.068 | 0.601 | 0.054 | 0.677 |
| MLF-L | 0.125 | 0.264 | 0.028 | 0.805 | 0.078 | 0.511 | 0.078 | 0.504 |
| MLF-R | 0.210 | 0.056 | 0.182 | 0.099 | 0.103 | 0.370 | 0.196 | 0.083 |

Abbreviations: AR, acoustic radiation; OR, optic radiation; FAT, Frontal aslant tract; MLF, medial longitudinal fasciculus; AVLT-D, Auditory Verbal Learning Test immediate recall; AVLT-D, Auditory Verbal Learning Test delay recall; AVLT-R, Auditory Verbal Learning Test recognition recall; L, left; R, right. All analyses were adjusted for age, sex, and education.

Table S10. Associations between the white matter tracts integrity and ERP metrics.

|  | **Encoding** | | | |  | **Retention** | |  | **Retrieval** | | | |
| --- | --- | --- | --- | --- | --- | --- | --- | --- | --- | --- | --- | --- |
|  | **N170** | | **P2** | |  | **NSW** | |  | **P1**  **(right occipital)** | | **N170** | |
|  | **β** | ***P*** | **β** | ***P*** |  | **β** | ***P*** |  | **β** | ***P*** | **β** | ***P*** |
| WMH | 0.320 | **0.004** | -0.159 | 0.162 |  | 0.359 | **0.001** |  | -0.024 | 0.838 | -0.211 | 0.068 |
| AF-L | -0.301 | **0.006** | 0.191 | 0.091 |  | -0.312 | **0.005** |  | 0.012 | 0.922 | 0.161 | 0.162 |
| AF-R | -0.382 | **<0.001** | 0.168 | 0.136 |  | -0.243 | **0.029** |  | 0.030 | 0.799 | 0.187 | 0.104 |
| CG-L | -0.365 | **0.001** | 0.171 | 0.141 |  | -0.312 | **0.006** |  | -0.068 | 0.577 | 0.194 | 0.101 |
| CG-R | -0.341 | **0.002** | 0.126 | 0.274 |  | -0.280 | **0.013** |  | -0.104 | 0.383 | 0.150 | 0.201 |
| IFOF-L | -0.332 | **0.003** | 0.126 | 0.278 |  | -0.312 | **0.006** |  | -0.055 | 0.654 | 0.132 | 0.267 |
| IFOF-R | -0.359 | **0.001** | 0.111 | 0.337 |  | -0.236 | **0.038** |  | 0.019 | 0.871 | 0.168 | 0.150 |
| ILF-L | -0.306 | **0.006** | 0.110 | 0.339 |  | -0.313 | **0.005** |  | -0.010 | 0.933 | 0.125 | 0.286 |
| SLF-L | -0.321 | **0.005** | 0.193 | 0.101 |  | -0.341 | **0.003** |  | 0.001 | 0.998 | 0.168 | 0.162 |
| SLF-R | -0.417 | **<0.001** | 0.141 | 0.225 |  | -0.349 | **0.002** |  | 0.024 | 0.840 | 0.202 | 0.086 |
| UF-L | -0.346 | **0.002** | 0.281 | **0.014** |  | -0.293 | **0.010** |  | 0.061 | 0.617 | 0.215 | 0.067 |
| CC | -0.393 | **0.001** | 0.131 | 0.278 |  | -0.317 | **0.008** |  | 0.031 | 0.805 | 0.185 | 0.133 |

Abbreviations: ERP, event-related potential; WMH, white matter hyperintensities; AF, arcuate fasciculus; CG, cingulum; IFOF, inferior fronto-occipital fasciculus; ILF, inferior longitudinal fasciculus; SLF, superior longitudinal fasciculus; UF, uncinate fasciculus; CC, corpus callosum; L, left; R, right. Analyses were adjusted for age, sex, and education. Bold type indicates statistical significance (*P* < 0.05).

Table S11. Associations between the white matter tracts integrity and neural oscillations.

|  | **ERSP (Encoding)** | | | |  | **ERSP (Retention)** | |  | **ERSP (Retrieval)** | | | |  | **ITPC (Encoding)** | |
| --- | --- | --- | --- | --- | --- | --- | --- | --- | --- | --- | --- | --- | --- | --- | --- |
|  | **Theta**  **(fronto-central)** | | **Theta**  **(occipital)** | |  | **Alpha**  **(right occipital)** | |  | **Theta**  **(fronto-central)** | | **Theta**  **(left occipital)** | |  | **Theta**  **(occipital)** | |
|  | **β** | ***P*** | **β** | ***P*** |  | **β** | ***P*** |  | **β** | ***P*** | **β** | ***P*** |  | **β** | ***P*** |
| WMH | -0.123 | 0.284 | -0.185 | 0.106 |  | 0.130 | 0.250 |  | -0.079 | 0.491 | -0.180 | 0.119 |  | -0.279 | **0.015** |
| AF-L | 0.046 | 0.684 | 0.185 | 0.105 |  | -0.061 | 0.582 |  | 0.075 | 0.510 | 0.164 | 0.155 |  | 0.357 | **0.001** |
| AF-R | 0.189 | 0.094 | 0.270 | **0.017** |  | -0.059 | 0.594 |  | 0.239 | **0.034** | 0.280 | **0.014** |  | 0.332 | **0.003** |
| CG-L | 0.064 | 0.582 | 0.130 | 0.269 |  | 0.008 | 0.943 |  | 0.039 | 0.737 | 0.124 | 0.295 |  | 0.280 | **0.017** |
| CG-R | 0.116 | 0.313 | 0.170 | 0.141 |  | -0.011 | 0.922 |  | 0.094 | 0.414 | 0.159 | 0.174 |  | 0.256 | **0.026** |
| IFOF-L | 0.075 | 0.522 | 0.181 | 0.121 |  | 0.032 | 0.781 |  | 0.101 | 0.390 | 0.158 | 0.183 |  | 0.302 | **0.009** |
| IFOF-R | 0.249 | **0.029** | 0.311 | **0.006** |  | 0.037 | 0.743 |  | 0.279 | **0.014** | 0.282 | **0.015** |  | 0.305 | **0.008** |
| ILF-L | 0.091 | 0.430 | 0.149 | 0.199 |  | 0.019 | 0.865 |  | 0.095 | 0.410 | 0.146 | 0.214 |  | 0.304 | **0.008** |
| SLF-L | 0.015 | 0.899 | 0.143 | 0.232 |  | -0.066 | 0.569 |  | 0.099 | 0.408 | 0.116 | 0.339 |  | 0.335 | **0.004** |
| SLF-R | 0.183 | 0.116 | 0.329 | **0.004** |  | -0.056 | 0.623 |  | 0.227 | 0.050 | 0.319 | **0.006** |  | 0.382 | **0.001** |
| UF-L | 0.062 | 0.597 | 0.198 | 0.089 |  | -0.042 | 0.715 |  | 0.078 | 0.503 | 0.150 | 0.204 |  | 0.269 | **0.021** |
| CC | 0.144 | 0.237 | 0.226 | 0.062 |  | -0.055 | 0.642 |  | 0.151 | 0.214 | 0.181 | 0.141 |  | 0.299 | **0.014** |

Abbreviations: ERSP, event-related spectral perturbation; ITPC, inter-trial phase coherence; WMH, white matter hyperintensities; AF, arcuate fasciculus; CG, cingulum; IFOF, inferior fronto-occipital fasciculus; ILF, inferior longitudinal fasciculus; SLF, superior longitudinal fasciculus; UF, uncinate fasciculus; CC, corpus callosum; L, left; R, right. All analyses were adjusted for age, sex, and education. Bold values indicate statistical significance (*P* < 0.05).

Table S12. Associations between the white matter tracts integrity and theta-gamma PAC.

|  | **Encoding PAC** | | | |  | **Retention PAC** | |  | **Retrieval PAC** | |
| --- | --- | --- | --- | --- | --- | --- | --- | --- | --- | --- |
|  | Frontal | | Left parietal | |  | Left temporal parietal | |  | Left frontal | |
|  | **β** | ***P*** | **β** | ***P*** |  | **β** | ***P*** |  | **β** | ***P*** |
| WMH | -0.470 | **< 0.001** | -0.185 | 0.137 |  | -0.122 | 0.329 |  | -0.299 | **0.015** |
| AF-L | 0.409 | **< 0.001** | -0.006 | 0.959 |  | -0.001 | 0.995 |  | 0.051 | 0.665 |
| AF-R | 0.379 | **0.001** | 0.034 | 0.772 |  | -0.103 | 0.379 |  | 0.060 | 0.609 |
| CG-L | 0.405 | **0.001** | 0.116 | 0.335 |  | -0.006 | 0.960 |  | 0.201 | 0.095 |
| CG-R | 0.463 | **< 0.001** | 0.121 | 0.311 |  | -0.050 | 0.676 |  | 0.209 | 0.078 |
| IFOF-L | 0.389 | **0.001** | 0.122 | 0.313 |  | 0.083 | 0.494 |  | 0.220 | 0.066 |
| IFOF-R | 0.342 | **0.003** | 0.076 | 0.527 |  | -0.081 | 0.498 |  | 0.124 | 0.298 |
| ILF-L | 0.336 | **0.004** | 0.141 | 0.237 |  | 0.107 | 0.371 |  | 0.194 | 0.103 |
| SLF-L | 0.456 | **< 0.001** | 0.022 | 0.857 |  | -0.005 | 0.967 |  | 0.095 | 0.441 |
| SLF-R | 0.470 | **< 0.001** | 0.151 | 0.209 |  | -0.083 | 0.490 |  | 0.174 | 0.147 |
| UF-L | 0.447 | **< 0.001** | 0.038 | 0.753 |  | 0.033 | 0.784 |  | 0.184 | 0.125 |
| CC | 0.486 | **< 0.001** | 0.047 | 0.706 |  | -0.046 | 0.716 |  | 0.187 | 0.136 |

Abbreviations: PAC, phase–amplitude coupling; ERSP, event-related spectral perturbation; ITPC, inter-trial phase coherence; WMH, white matter hyperintensities; AF, arcuate fasciculus; CG, cingulum; IFOF, inferior fronto-occipital fasciculus; ILF, inferior longitudinal fasciculus; SLF, superior longitudinal fasciculus; UF, uncinate fasciculus; CC, corpus callosum; L, left; R, right. Electrodes were selected as follows: frontal (E11, E13, E124) and left parietal (E42, E47, E52) for encoding, left temporo-parietal (E50) for retention, and left frontal (E18) for retrieval. All analyses were adjusted for age, sex, and education. Bold values indicate statistical significance (*P* < 0.05).

Table S13. Association between white matter tracts integrity and memory performance.

|  | **Accuracy** | | **RT** | | **MOCA** |  | **AVLT-D** |  | **AVLT-R** |  |
| --- | --- | --- | --- | --- | --- | --- | --- | --- | --- | --- |
|  | **β** | ***P*** | **β** | ***P*** | **β** | ***P*** | **β** | ***P*** | **β** | ***P*** |
| WMH | -0.375 | **< 0.001** | 0.262 | **0.015** | -0.278 | **0.013** | -0.169 | 0.152 | -0.244 | 0.037 |
| AF-L | 0.267 | **0.014** | -0.343 | **0.001** | 0.241 | **0.030** | 0.203 | 0.080 | 0.198 | 0.083 |
| AF-R | 0.097 | 0.379 | -0.275 | **0.010** | 0.193 | 0.083 | 0.149 | 0.198 | 0.203 | 0.076 |
| CG-L | 0.391 | **< 0.001** | -0.355 | **0.001** | 0.286 | **0.012** | 0.313 | **0.008** | 0.304 | **0.009** |
| CG-R | 0.357 | **0.001** | -0.379 | **< 0.001** | 0.291 | **0.009** | 0.296 | **0.011** | 0.308 | **0.007** |
| IFOF-L | 0.377 | **0.001** | -0.348 | **0.001** | 0.307 | **0.007** | 0.250 | **0.034** | 0.234 | **0.045** |
| IFOF-R | 0.114 | 0.308 | -0.283 | **0.009** | 0.324 | **0.004** | 0.194 | 0.098 | 0.090 | 0.443 |
| ILF-L | 0.289 | **0.008** | -0.262 | **0.016** | 0.274 | **0.015** | 0.233 | **0.047** | 0.311 | **0.007** |
| SLF-L | 0.317 | **0.005** | -0.357 | **0.001** | 0.241 | **0.038** | 0.281 | **0.019** | 0.312 | **0.008** |
| SLF-R | 0.189 | 0.092 | -0.300 | **0.006** | 0.236 | **0.038** | 0.266 | **0.024** | 0.269 | **0.020** |
| UF-L | 0.338 | **0.002** | -0.386 | **< 0.001** | 0.215 | 0.059 | 0.174 | 0.143 | 0.163 | 0.164 |
| CC | 0.305 | **0.008** | -0.354 | **0.002** | 0.299 | **0.011** | 0.276 | **0.025** | 0.248 | **0.041** |

Abbreviations: WMH, white matter hyperintensities; RT, reaction time; MoCA, Montreal Cognitive Assessment; AVLT-D, Auditory Verbal Learning Test delay recall; AVLT-R, Auditory Verbal Learning Test recognition recall; AF, arcuate fasciculus; CG, cingulum; IFOF, inferior fronto-occipital fasciculus; ILF, inferior longitudinal fasciculus; SLF, superior longitudinal fasciculus; UF, uncinate fasciculus; CC, corpus callosum; L, left; R, right. All analyses were adjusted for age, sex, and education. Bold values indicate statistical significance (*P* < 0.05).

Table S14. Mediation analysis of the association between electrophysiological markers and cognitive performance (MoCA).

|  |  | **Total Effect** |  | **Direct Effect** |  | **Indirect Effect** |  | **Proportion Mediated** |
| --- | --- | --- | --- | --- | --- | --- | --- | --- |
|  |  | Estimation  (95% CI) | *P* | Estimation  (95% CI) | *P* | Estimation  (95% CI) | *P* | Estimation |
| **ERSP** |  |  |  |  |  |  |  |  |
| ***Encoding*** |  |  |  |  |  |  |  |  |
| Theta (occipital)  (MoCA) | IFOF-R | 0.323 (0.109-0.536) | **0.002** | 0.246 (0.021-0.473) | **0.028** | 0.077 (0.006-0.185) | **0.027** | 22.72% |
|  | SLF-R | 0.235 (0.015-0.454) | **0.043** | 0.144 (-0.087-0.379) | 0.213 | 0.091 (0.011-0.208) | **0.013** | 37.05% |
| **ITPC** |  |  |  |  |  |  |  |  |
| ***Encoding*** |  |  |  |  |  |  |  |  |
| Theta (occipital) (MoCA) | IFOF-L | 0.335 (0.111-0.562) | **0.004** | 0.258 (0.029-0.487) | **0.028** | 0.077 (0.006-0.185) | **0.023** | 22.23% |
|  | IFOF-R | 0.349 (0.130-0.568) | **0.002** | 0.269 (0.041-0.496) | **0.016** | 0.079 (0.007-0.187) | **0.017** | 21.95% |
|  | ILF-L | 0.306 (0.082-0.529) | **0.009** | 0.228 (-0.002-0.456) | 0.052 | 0.078 (0.006-0.188) | **0.022** | 24.37% |
|  | SLF-L | 0.279 (0.050-0.512) | **0.022** | 0.191 (-0.049-0.429) | 0.110 | 0.088 (0.009-0.205) | **0.012** | 30.03% |
|  | SLF-R | 0.273 (0.039-0.505) | **0.026** | 0.161 (-0.087-0.409) | 0.185 | 0.112 (0.018-0.239) | **0.009** | 39.73% |
|  | CC | 0.305 (0.068-0.545) | **0.016** | 0.218 (-0.019-0.461) | 0.076 | 0.087 (0.008-0.206) | **0.018** | 27.32% |

Abbreviations: ERSP, event-related spectral perturbation; ITPC, inter-trial phase coherence; MoCA, Montreal Cognitive Assessment; AVLT-D, Auditory Verbal Learning Test delay recall; RT: reaction time; IFOF, inferior fronto-occipital fasciculus; ILF, inferior longitudinal fasciculus; SLF, superior longitudinal fasciculus; CC, corpus callosum; L, left; R, right. All analyses were adjusted for age, sex, and education. Bold values indicate statistical significance (*P* < 0.05).

Table S15. Predictive value of electrophysiological markers.

|  | **AUC** | **95% CI** | **Spens** | **Spec** |
| --- | --- | --- | --- | --- |
| WMH | 0.944 | 0.899-0.988 | 84.4% | 92.1% |
| EEG | 0.947 | 0.901-0.992 | 91.1% | 92.1% |

ROC analysis for the prognostic value (measured by EEG biomarkers) of various biomarker combinations for the two populations. Examined EEG biomarkers included the theta and alpha oscillatory power, theta ITPC, and theta–gamma PAC. All models were adjusted for age, sex, and education. Abbreviations: AUC, Area Under the Curve; CI, Confidence Interval.

Table S16. Group differences in lacunes, perivascular spaces, and cerebral microbleeds between NC and msWMH.

|  | **NC (n = 38)** | **msWMH (n = 45)** | **t/χ^2^** | ***P*** |
| --- | --- | --- | --- | --- |
| Lacunes, yes (%) | 2 (5.3) | 9 (20.0) | 2.716 | 0.099 |
| PVS volume (mm³) | 802.237 (395.907) | 1605.341 (603.282) | -7.004 | **< 0.001** |
| CMBs, yes (%) | 1 (2.6) | 8 (17.8) | 3.537 | 0.060 |

Continuous variables are presented as mean ± standard deviation (SD), and categorical variables as n (%). Group differences were compared using independent-sample t-tests for continuous variables and χ^2^ tests for categorical variables. Abbreviations: PVS, perivascular spaces; CMBs, cerebral microbleeds.

Table S17. Associations between lacunes, perivascular spaces, cerebral microbleeds, and WMH burden.

|  | **WMH** | |
| --- | --- | --- |
|  | **ρ** | ***P*** |
| lacunes | 0.154 | 0.164 |
| PVS | 0.648 | **< 0.001** |
| CMBs | 0.202 | 0.084 |

Abbreviations: WMH, white matter hyperintensities; PVS, perivascular spaces; CMBs, cerebral microbleeds.

Table S18. Associations between lacunes, perivascular spaces, cerebral microbleeds, and EEG metrics.

|  |  | **PVS** | |
| --- | --- | --- | --- |
|  |  | **β** | ***P*** |
| **ERP** |  |  |  |
| ***Encoding*** | N170 | -0.154 | 0.163 |
|  | P2 | 0.186 | 0.090 |
| ***Retention*** | NSW | 0.172 | 0.125 |
| ***Retrieval*** | P1 (right occipital) | -0.173 | 0.131 |
|  | P2 | -0.300 | **0.007** |
| **ERSP** | | | |
| ***Encoding*** | Theta (fronto-central) | -0.236 | **0.033** |
|  | Theta (occipital) | -0.177 | 0.109 |
| ***Retention*** | Alpha (right occipital) | 0.148 | 0.177 |
| ***Retrieval*** | Theta (fronto-central) | -0.189 | 0.087 |
|  | Theta (left occipital) | -0.227 | **0.042** |
| **ITPC** | | | |
| ***Encoding*** | Theta (occipital) | -0.234 | **0.035** |
| **PAC** | | | |
| ***Encoding*** | Frontal | -0.429 | **< 0.001** |
|  | Left parietal | -0.203 | 0.078 |
| ***Retention*** | Left temporal parietal | -0.231 | **0.046** |
| ***Retrieval*** | Left frontal | -0.146 | 0.211 |

Abbreviations: PVS, perivascular spaces; PAC, phase–amplitude coupling; ERSP, event-related spectral perturbation; ITPC, inter-trial phase coherence; WMH, white matter hyperintensities. Electrodes of PAC were selected as follows: frontal (E11, E13, E124) and left parietal (E42, E47, E52) for encoding, left temporo-parietal (E50) for retention, and left frontal (E18) for retrieval. All analyses were adjusted for age, sex, and education. Bold values indicate statistical significance (*P* < 0.05).

Table S19. Associations between perivascular spaces and cognitive.

|  | **PVS** | |
| --- | --- | --- |
|  | **β** | ***P*** |
| **Accuracy** | 0.006 | 0.958 |
| **RT** | 0.012 | 0.909 |
| **MoCA** | -0.137 | 0.212 |
| **AVLT-I** | -0.040 | 0.715 |
| **AVLT-D** | -0.118 | 0.299 |
| **AVLT-R** | -0.149 | 0.188 |
| **DST-F** | -0.183 | 0.103 |
| **DST-B** | -0.180 | 0.109 |
| **TMTB-A** | 0.083 | 0.465 |

Abbreviations: PVS, perivascular spaces; RT, reaction time; MoCA, Montreal Cognitive Assessment; AVLT-I, Auditory Verbal Learning Test immediate recall; AVLT-D, Auditory Verbal Learning Test delayed recall; AVLT-R, Auditory Verbal Learning Test recognition recall; DST-F/B, Digit Span Test forward and backward; TMT, Trail Making Test. Analyses were adjusted for age, sex, and education. Bold type indicates statistical significance (*P* < 0.05).

1. Boutinaud P, Tsuchida A, Laurent A, et al. 3D Segmentation of Perivascular Spaces on T1-Weighted 3 Tesla MR Images With a Convolutional Autoencoder and a U-Shaped Neural Network. *Front Neuroinform*. 2021;15:641600. doi:10.3389/fninf.2021.641600

2. Li H, Jacob MA, Cai M, et al. Perivascular Spaces, Diffusivity Along Perivascular Spaces, and Free Water in Cerebral Small Vessel Disease. *Neurology*. 2024;102(9):e209306. doi:10.1212/wnl.0000000000209306

3. Wardlaw JM, Smith EE, Biessels GJ, et al. Neuroimaging standards for research into small vessel disease and its contribution to ageing and neurodegeneration. *Lancet Neurol*. 2013;12(8):822-838. doi:10.1016/s1474-4422(13)70124-8

4. Duering M, Biessels GJ, Brodtmann A, et al. Neuroimaging standards for research into small vessel disease-advances since 2013. *Lancet Neurol*. 2023;22(7):602-618. doi:10.1016/s1474-4422(23)00131-x
